# Ferro-self-assembly: magnetic and electrochemical adaptation of a multiresponsive zwitterionic metalloamphiphile showing a shape-hysteresis effect†

**DOI:** 10.1039/d0sc05249c

**Published:** 2020-11-03

**Authors:** Stefan Bitter, Moritz Schlötter, Markus Schilling, Marina Krumova, Sebastian Polarz, Rainer F. Winter

**Affiliations:** Department of Chemistry, University of Konstanz Universitätsstrasse 10 78457 Konstanz Germany rainer.winter@uni-konstanz.de; Institute of Inorganic Chemistry, Leibniz-University Hannover Callinstrasse 9 30167 Hannover Germany sebastian.polarz@aca.uni-hannover.de

## Abstract

Metallosurfactants are molecular compounds which combine the unique features of amphiphiles, like their capability of self-organization, with the peculiar properties of metal complexes like magnetism and a rich redox chemistry. Considering the high relevance of surfactants in industry and science, amphiphiles that change their properties on applying an external trigger are highly desirable. A special feature of the surfactant reported here, 1-(*Z*)-heptenyl-1′-dimethylammonium-methyl-(3-sulfopropyl)ferrocene (6), is that the redox-active ferrocene constituent is in a gemini-position. Oxidation to 6^+^ induces a drastic change of the surfactant's properties accompanied by the emergence of paramagnetism. The effects of an external magnetic field on vesicles formed by 6^+^ and the associated dynamics were monitored *in situ* using a custom-made optical birefringence and dual dynamic light scattering setup. This allowed us to observe the optical anisotropy as well as the anisotropy of the diffusion coefficient and revealed the field-induced formation of oriented string-of-pearls-like aggregates and their delayed disappearance after the field is switched off.

## Abstract 2

The self-organization properties of a stimuli responsive amphiphile can be altered by subjecting the paramagnetic oxidized form to a magnetic field of 0.8 T and monitored in real time by coupling optical birefringence with dynamic light scattering.

## Graphical abstract

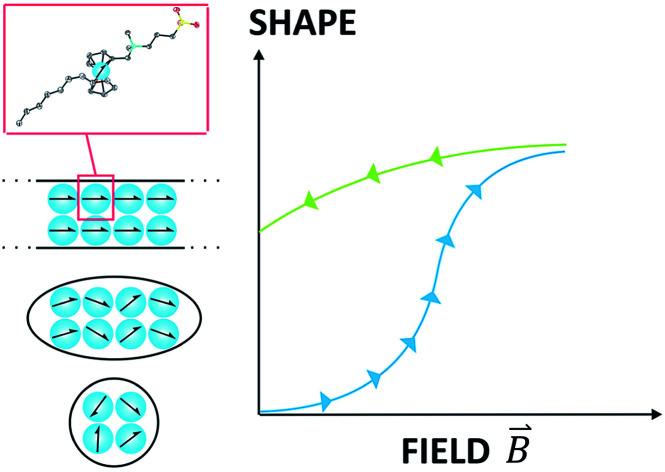

Amphiphiles (or surfactants) combine hydrophilic (the so-called headgroups) and lipophilic entities (the so-called tails) as integral parts of their molecular structures. This particular construction principle provides them with the ability to display concentration-dependent self-organization in nonpolar and polar solvents.^1^ Amphiphiles with advanced functions that go far beyond the traditional ones as emulsifiers, stabilizing agents for interfaces, or detergents were meanwhile realized by skillful manipulation of any of its constituents.^2–4^ Recent examples are micellar LEDs,^5,6^ catalysts,^7–9^ or batteries.^10^ Such applications are important hallmarks on the way to even more sophisticated amphiphiles such as the ones found in nature, *e.g.* in the pockets of enzymes.^11–18^ An important milestone is the advent of (multi-) stimuli-responsive amphiphiles, whose encoded functionalities respond to (different) external triggers. Such systems are capable of adaptive self-assembly, which can be controlled using an external input such as the pH, temperature, ionic strength, or redox state.^19–26^

Paramagnetic amphiphiles, recently reviewed by Eastoe and coworkers, constitute a fascinating family of stimuli-responsive surfactants.^27^ Particular attention has been paid to magnetic ionic liquids based on amphiphilic transition metal complexes, as their properties are often superior to those of conventional magnetic fluids (ferrofluids).^28–31^ Self-assembly results in high effective concentrations of the paramagnetic metal centers, and this in turn allows us to control their physico-chemical properties and the morphologies of their superstructures through an external magnetic field. Such a scheme has the added advantage that the external stimulus is non-invasive. In many current realizations of such systems, however, the magneto-active (transition) metal ion is only present as a constituent of the counterion of a cationic surfactant, but is not an integral constituent of the surfactant itself.^21,30,31^

Some of us have previously reported redox-switchable as well as paramagnetic stimuli-responsive amphiphiles of relevance to the current work.^32,33^ We thought that ferrocene would be an ideal building block in order to combine both these kinds of stimuli within one single amphiphile.^34–37^ On oxidation, the diamagnetic, hydrophobic ferrocene nucleus is transformed into a paramagnetic *S* = 1/2 ferrocenium ion with a distinct hydrophilic character.^38–41^ Oxidation does hence not only generate a magnetic moment, but also transfers the ferrocene nucleus from the lipo- to the hydrophilic part of the amphiphile, thereby changing its entire structure. A 1,1′-disubstitution pattern of the ferrocene scaffold, which is synthetically well accessible,^34,42–44^ seemed particularly suited for such an endeavor.

Studies on paramagnetic amphiphiles are often thwarted by the non-trivial analytics involved in their characterization. Detailed investigations often rely on small-angle neutron scattering (SANS), which is time-consuming and costly and suffers from poor availability.^27,30,31,45–47^ Moreover, SANS is only of limited value for following kinetically fast processes which would be desirable for the live monitoring of structural changes occurring in solution. Optical birefringence is a well-established method to monitor the dynamic response of materials to external fields.^48–50^ Although of high intrinsic value, optical birefringence measurements in magnetic fields were only rarely applied for the study of paramagnetic amphiphiles.^29^

We here report the zwitterionic, ferrocene-based amphiphile FcNMe_2_SO_3_Heptene 6 (see Fig. 1, Fc = ferrocenyl) with a sultone headgroup. Compound 6 is unique in that its self-assembly properties can be controlled by three different external stimuli, namely the (i) addition of an electrolyte, (ii) addition of an oxidant/reductant, and (iii) exposure to an external magnetic field. We also demonstrate that optical birefringence in combination with dynamic light scattering (DLS) measurements in two orthogonal directions provides detailed insights into the functional response of aggregated magnetic nanoparticles formed by 6^+^ to an external magnetic field in real time. Specifically, we have observed the formation of string-of-pearls-like aggregates of 6^+^ in a magnetic field (0.8 T), the field-induced anisotropy of the diffusion of aggregated nanoparticles, and a hysteresis effect for their disappearance after the magnetic field is switched off. Thus, the anisotropy of larger aggregates persists for more than 5 min, while the structural alignment of smaller ones vanishes at a significantly faster rate.

**Fig. 1:**
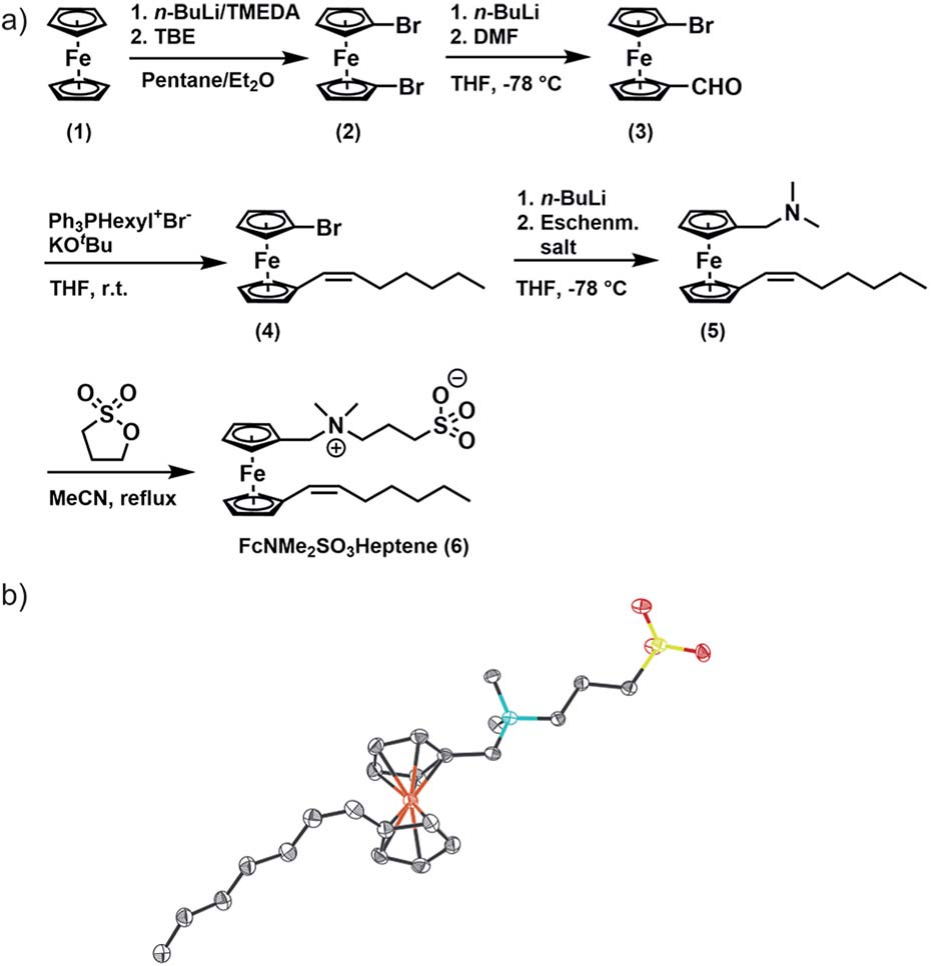
Synthesis of FcNMe_2_SO_3_Heptene (6). (a) Synthesis of 6; (b) molecular structure of 6 crystallized from acetonitrile. C; dark grey, N; turquoise, Fe; orange, S; yellow, O; red, H atoms are omitted for clarity.

## Results

### Synthesis and characterization of FcNMe_2_SO_3_Heptene (6)

FcNMe_2_SO_3_Heptene was obtained in four consecutive synthetic steps from literature-known 1,1′-dibromoferrocene (2) (Fig. 1).^51^ Formylation of 2 yields asymmetrically functionalized 1-formyl-1′-bromoferrocene (3). 3 was then subjected to a Wittig reaction with hexyltriphenylphosphonium bromide to provide 1-(*Z*)-heptenyl-1′-bromoferrocene (4).^52–54^ Subsequent conversion with Eschenmoser's salt under Mannich-like conditions^55^ followed by quaternization with 1,3-propane sultone ultimately yielded the target compound 6.^56^

6 was fully characterized by ^1^H and ^13^C{^1^H} NMR spectroscopy, electrospray ionization mass spectrometry (ESI MS), IR spectroscopy, UV-Vis spectroscopy and X-ray crystallography. Fig. S1(a–g) of the ESI† provide graphical representations of the corresponding spectra and the assignments of IR peaks. The ESI MS signals and their isotope patterns are in perfect agreement with the simulated ones for the M^+^ and (M + H)^+^ peaks of 6. Of particular note is the observation of intense mass peaks corresponding to a dimer of 6 and to higher oligomers up to the tetramer as well as their Na^+^ adducts. This is a token of the high propensity of 6 to aggregate. UV-Vis absorption spectra recorded in acetonitrile show an intense π → π* absorption band at *λ*_max_ = 284 nm and a weaker, characteristic band of the d_*δ*_ → d_π_/Cp_π_ excitation of a ferrocene species (the HOMO → LUMO transition) at *λ*_max_ = 444 nm (*ε* = 225 M^−1^ cm^−1^), which accounts for the pale orange color of 6.^57–59^ The respective DFT calculated frontier MOs of 6 are shown in Fig. S2 of the ESI.†

### Surfactant properties of 6 in the presence and absence of added salts

**Fig. 2:**
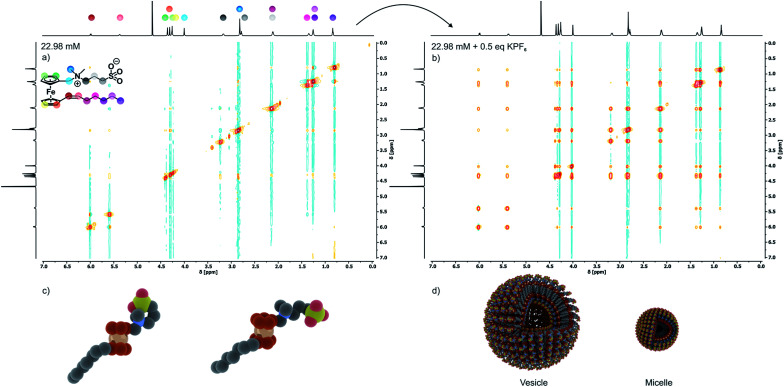
Triggered aggregation of compound 6 by the addition of an external electrolyte. (a) NOESY spectrum of 6 (22.98 mM) in pure D_2_O; (b) NOESY spectrum of 6 (22.98 mM) in 11.5 mM KPF_6_ (0.5 eq.) in D_2_O; (c) graphical illustrations of the closed (left) and open (right) headgroup conformations of 6; (d) graphical illustrations of aggregates (micelle and vesicle).

As is known for other sulfobetaine-containing polymers,^60^ the solubility and the preferred conformation of 6 are highly sensitive to the solvent polarity and the kind and concentration of an added electrolyte. In nonpolar solvents, 6 undergoes intramolecular ion pairing by backfolding of the C_3_H_6_SO_3_^−^ moiety onto the ammonium part of the headgroup as was verified by NOESY experiments in CDCl_3_ (Fig. S3a of the ESI†). In spite of its zwitterionic sulfobetaine headgroup, 6 is only sparingly soluble in pure water, already saturating at a concentration of 0.87 mM. Under these conditions, 6 adopts an open configuration as indicated by the absence of NOESY cross peaks between the methylene protons of the propylene connector and the methyl and methylene protons of the Fc-CH_2_NMe_2_ segment (see Fig. 2a). In this open configuration, individual molecules of 6 associate with neighboring molecules by intermolecular ion pairing between the oppositely charged ammonium and sulfonate groups, thereby annihilating the individual charges. This accounts for the low water solubility of 6.

This conformation and intermolecular ion pairing are preserved in the solid state. Packing diagrams in Fig. S4 of the ESI† show that, in the crystal, molecules of 6 associate with four neighboring molecules *via* close interionic contact O⋯N of 3.764 Å to 4.098 Å between the NR_4_^+^ and the RSO_3_^−^ parts of the headgroups.

These interionic interactions are supported by a total of 10 strong, pairwise hydrogen bonds C–H⋯O between hydrogen atoms at the NMe groups or at a cyclopentadienide ligand and the sulfonate oxygen atoms. The latter range from 2.187 Å, which is as much as 0.533 Å shorter than the sum of their van der Waals radii, to 2.527 Å. Additional hydrophobic interactions between the oleophilic *Z*-heptenyl chains organize crystalline 6 into a lamellar structure resulting from head-to-head/tail-to-tail arrangements of individual molecules. SAXS and PXRD measurements of solid samples of 6 obtained from aqueous solutions show a microcrystalline pattern with *d*_100_ = 2.68 nm and the corresponding higher-order reflexes (see Fig. S5 of the ESI†). The observed PXRD pattern matches with that calculated from the experimental X-ray structure, which proves that intermolecular ion pairing with an alignment of the oppositely charged headgroups prevails under these conditions.

Disruption of this packing motif and the typical foam formation of an amphiphile are, however, observed after the addition of KPF_6_ or KNO_3_ to aqueous solutions of 6. According to the Hofmeister salt series, large and polarizable ions, in particular anions, have the largest effect (Fig. S7†).^60,61^ Aggregation upon electrolyte addition can be monitored *via* NOESY (Fig. 2b) and DOSY (Fig. S6 of the ESI†) measurements and is further supported by the SAXS and PXRD data (see Fig. S5 of the ESI†). Ion-pairing of the ammonium and sulfonate entities of the sultone headgroup with the appropriate ions of an external electrolyte breaks the intermolecular ionic interactions. As a consequence, the solubility of 6 in water increases 26 fold, from 0.87 mM to 23 mM, with a concomitant change of the zeta potential from +50 mV to −49 mV.

**Fig. 3:**
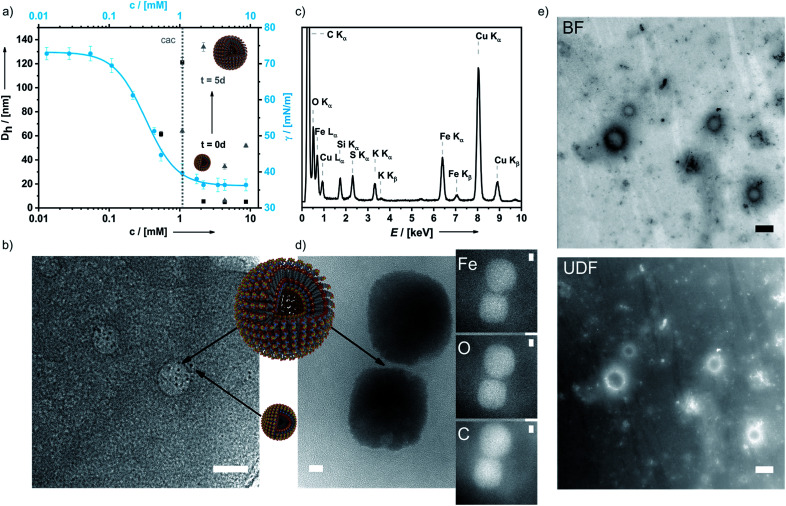
Surfactant properties of 6. (a) Concentration-dependent surface tension and DLS measurements of 6 at *t* = 0 d and after 5 d; (b) cryo-TEM image of 6 (micelles and vesicles are denoted by black arrows); (c) area-selected EDX results of 6; (d) area-selected ESI images (TEM) of a dried sample of 6 for Fe, O and C; (e) BF and UDF measurements in the STEM mode proving the presence of heavy atoms in the double layer membranes of the vesicles. Scale bar: 25 nm.

In aqueous 0.1 M KNO_3_ or 0.01 M KPF_6_ solutions compound 6 shows remarkable surfactant properties and exhibits a surface activity *γ* of 36 mN m^−1^. The surface tension curve can be treated as a Gibbs isotherm, and a surface excess area *Γ* of 6.93 μmol m^−2^ and an average area *A*_m_ of 24 Å^2^ per molecule at the water–air interface are obtained. Dynamic light scattering (DLS) and surface tension measurements (Fig. 3a) provide a critical aggregation concentration *cac* of *ca.* 1.0 mM. At concentrations > *cac*, and immediately after preparation, aggregates with a size of *D*_H_ ≈ 4 nm form in both 0.1 M KNO_3_ and 0.01 M KPF_6_ (Fig. S8a and b†). Considering that 6 has a diameter of *ca.* 2.1 nm, one can infer that under these conditions spherical micelles are formed. After 5 days in solution, the size of the aggregates has increased to 40–120 nm in aqueous KNO_3_ and to 120–180 nm in aqueous KPF_6_. The *d*_hkl_ value of 3.26 nm obtained from SAXS/PXRD measurements corresponds to roughly twice the molecular length of 6. This is a typical feature of vesicles. Cryo-transmission electron microscopy (cryo-TEM) images consequently show large spherical aggregates, whose diameters correspond well with the DLS data, besides smaller micelles. Area-selected energy-dispersive X-ray spectroscopy (EDX), electron spectroscopic imaging (ESI), bright field (BF)/dark field (UDF) measurements (Fig. 3c–e), and electron energy loss spectroscopy (EELS) measurements in the scanning transmission electron microscopy (STEM) mode (Fig. S9 of the ESI†) confirm the presence of the respective elements (Fe, C, N, O, S and K) in the vesicle walls. At concentrations *c* ≈ 4.34 mM the further growth of the primary aggregates into vesicles is effectively suppressed as shown by the respective number distributions (Fig. S8c and d of the ESI†).

A temperature increase from 20 °C to 50 °C has no detectable effect on aggregate formation (Fig. S8e of the ESI†). At even higher concentrations, typical amphiphilic properties are preserved and lyotropic liquid crystals are formed (Fig. S8f of the ESI†). Hence, addition of an electrolyte converts intermolecular ion pairs or lamellar aggregates into micelles and vesicles and simultaneously increases the solubility of 6 26 fold.

### Redox-induced changes of the surfactant properties of 6

**Fig. 4:**
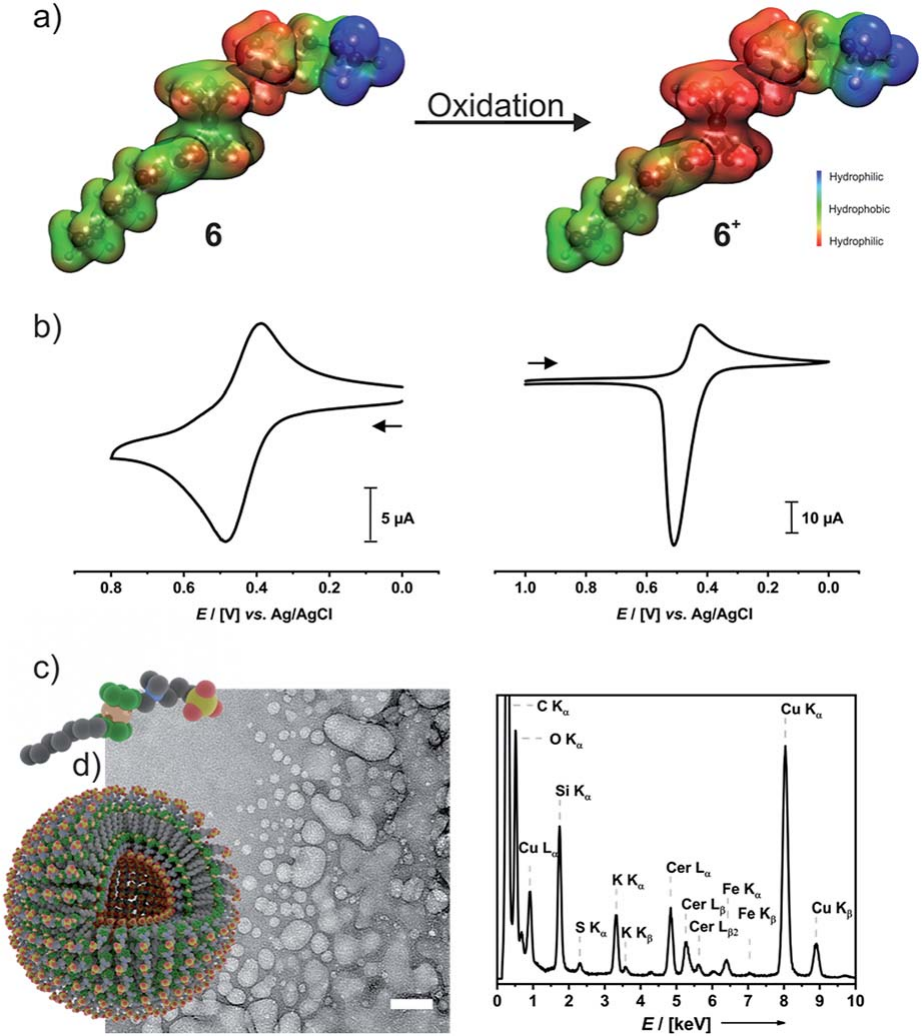
Redox-switchability of compound 6. (a) Electrostatic potential surfaces of 6 (left) and 6^+^ (right); (b) CVs of 6 (left) and 6^+^ (right) obtained in 0.1 M aqueous KNO_3_ with a sweep rate of 100 mV s^−1^; (c) cryo-TEM image of 6^+^ (left) and area-selected EDX spectrum (right); (d) graphical illustrations of the monomer and vesicles of 6^+^. Scale bar: 50 nm.

An important feature of ferrocenes is their propensity to undergo reversible one-electron oxidation. In the case of ferrocene-derived amphiphiles, the formerly hydrophobic ferrocene nucleus is transformed into a hydrophilic ferrocenium species and thus becomes part of the headgroup. The calculated electrostatic potential surfaces shown in Fig. 4a clearly demonstrate this.

Cyclic voltammograms (CVs) of 6 (left middle panel of Fig. 4b) and 6^+^ (right panel) indicate that the 6/6^+^ wave constitutes a chemically reversible redox couple with a half-wave potential *E*_1/2_ of 438 mV *versus* the Ag/AgCl reference. While the reduction peak of 6^+^ has the typical shape of a diffusion-controlled process, the sharp, symmetric anodic counterpeak indicates that electrogenerated 6 is adsorbed on the electrode surface. Concomitantly, the anodic forward peak in the CV of 6 is sharper and more intense than the cathodic return wave. This matches with earlier observations on ammonium-functionalized amphiphilic ferrocenes with a dodecyl chain at the ammonium nitrogen atom.^62,63^

Preparative oxidation of 6 was performed either chemically with cerium ammonium nitrate ((NH_4_)_2_[Ce(NO_3_)_6_], CAN) as the oxidant or by electrochemical means and is accompanied by a color change from yellow to green (Fig S10a of the ESI†). The UV-Vis absorption spectrum of 6^+^ in water features the typical ferrocenium absorptions at *λ*_max_ = 585 nm (*ε* = 220 M^−1^ cm^−1^) and at *λ*_max_ = 820 nm (*ε* = 382 M^−1^ cm^−1^) (Fig. S10b of the ESI†).^64,65^ Chemical (Na_2_S_2_O_3_) or electrochemical reduction converts 6^+^ back to the neutral state. The NMR spectra of a sample recovered after a full oxidation/reduction cycle indicate isomerization of the heptenyl tail from the *Z* to the *E* configuration (see Fig. S10c of the ESI†). This is, however, not expected to affect the amphiphilic properties.

In a 0.1 M aqueous KNO_3_ solution, 6^+^ still forms a foam. Concentration-dependent surface-tension measurements show a surface activity *γ* of 34 mN m^−1^, which is virtually identical to that of neutral 6 (Fig. S11a of the ESI†). The surface excess area *Γ* of 4.84 μmol m^−2^ is, however, *ca.* 30% smaller than that of 6 and corresponds to an average area *A*_m_ of 34 Å^2^ per molecule of 6^+^ at the water–air interface. DLS measurements as depicted in Fig. S11b of the ESI† indicate that aggregates of 6^+^ exhibit very broad size distributions while their average size gradually increases over time (see Fig. S11c of the ESI†). DLS yielded a *cac* of *ca.* 1.4 mM (Fig. S11d of the ESI†). The rather broad size distribution of the dispersed aggregates matches with the results of cryo-TEM measurements on a freshly prepared sample of 6^+^ (Fig. 4c). Individual spherical objects (vesicles) with double layer membranes of vastly different sizes and curvatures are observed besides more irregularly shaped and larger, fused vesicles as well as large quantities of smaller micelles. No thermodynamically favored morphology seems to prevail under these conditions. As the sulfonate moiety of 6^+^ can interact not only with the ammonium but also with the cationic ferrocenium moiety, 6^+^ may exist in several conformations which differ with respect to their effective headgroup sizes. This may lead to large variations in effective headgroup charges and areas and, hence, different packing parameters. Area-selected EDX measurements on chemically oxidized samples (Fig. 4c) prove the presence of the elements Fe, N, S, O, C, and Ce in these aggregates. EELS measurements (Fig. S12 of the ESI†) in the STEM mode agree with the EDX data. Hence, while oxidation does not have much of an influence on the typical surfactant properties of 6/6^+^, it inflicts drastic changes on their self-assembly properties and aggregation behavior in solution.

### The triggering of the surfactant properties of 6^+^ by magnetic fields

**Fig. 5:**
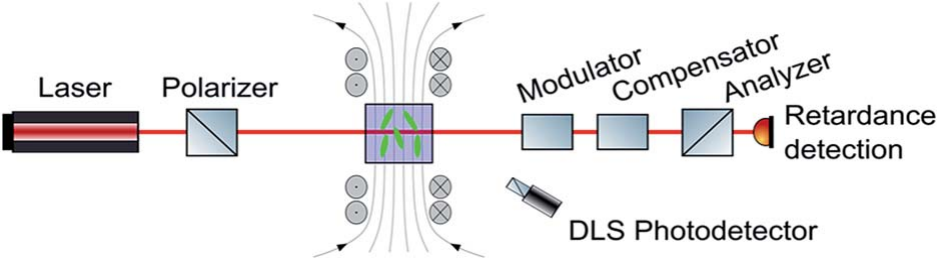
Schematic drawing of the setup to detect magnetic field-induced changes in DLS and birefringence.

Ferrocenium ions are associated with an effective magnetic moment *μ*_eff_ of 2.3–2.6 *μ*_B_.^38,39,65^ As the ferrocenium constituent is an integral part of the amphiphilic structure of 6^+^, an external magnetic field might exert a large effect on the morphologies of its aggregates. Seeking a method to monitor any such changes, we combined two DLS setups with optical birefringence detection inside a split-coil electromagnet. The additional dual DLS system allows for an independent determination of the diffusion coefficients *D*_‖_ and *D*_⊥_, parallel and perpendicular to the external field. The ratio *D*_‖_/*D*_⊥_ represents the effect of the structural alignment upon Brownian motion and strongly supports our interpretation of how the aggregates respond to an external magnetic field. The experimental setup is schematically shown in Fig. 5, and further details are provided in the Methods section.^48^

The following results will show that our approach is indeed capable of monitoring field-induced morphological changes in real time. Moreover, we were also able to show that large superstructures of aggregates of 6^+^ form and align parallel to the magnetic field when the field is switched on and reorient and/or disassemble with a sizable delay after the field is switched off.

Optical birefringence Δ*n* relies on the anisotropy of a molecular orientation distribution, according to eqn (1).

1 Δ*n* = *n*_‖_ − *n*_⊥_

*n*_‖_ is the refractive index for light that is polarized parallel to the external magnetic field *B*_ext_ and *n*_⊥_ corresponds to a polarization perpendicular to *B*_ext_. The external magnetic field imposes the energy Δ*χB*_ext_^2^, which in turn will act to deform any magnetic aggregates. The magnetic energy is counteracted by the thermal energy *kT* and the mechanical properties of the particles' membranes.

**Fig. 6:**
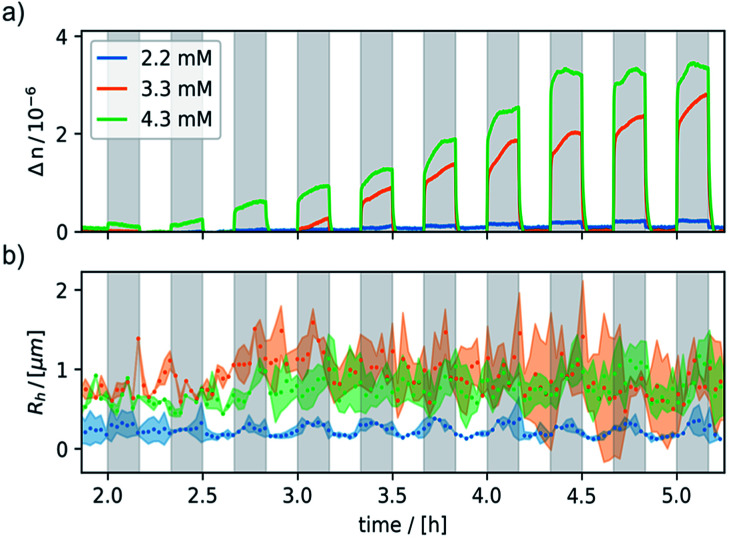
Magnetic field-induced (0.8 T) response of birefringence (top panel (a)) and of the hydrodynamic radius (bottom panel (b)). The *x*-axis indicates the time passed after oxidation. The field was switched on and off for 10 minutes, respectively. The shaded, grey areas denote the time during which the field was switched on (*B*_0_ = 0.8 T). (a) The magnetically induced optical birefringence; (b) effective hydrodynamic radius obtained by averaging the two DLS measurements. The dots represent the mean hydrodynamic radius *R*_h_. The width of the semi-transparent area at *R*_h_ is a measure of the polydispersity PDI, scaled by a factor of 0.2.

Negligible birefringence features (Δ*n* < 10^−7^) were obtained for neutral 6 and the oxidant CAN, indicating that the observed response is indeed due to 6^+^. The onset of a detectable response to the external magnetic field matched with the *cac* of ∼1.4 mM, thus showing that it is tied to the presence of aggregates of 6^+^. Under these conditions, an external magnetic field of 0.8 T was switched on and off for 10 min, respectively. Fig. 6 shows the data collected for three freshly prepared, differently concentrated samples of 6^+^ (*c* = 2.2, 3.3, and 4.3 mM). Magnetically induced optical birefringence can be observed for all three investigated samples, but with strongly differing intensities. The first indications of optical birefringence as a response to the applied external magnetic field appear *ca.* 2 h after oxidation of 6 to 6^+^. More substantial intensity increases are noted after *ca.* 3.5 h for the 2.2 mM sample, after *ca.* 3 h for an amphiphile concentration of 3.3 mM, and even after *ca.* 2.5 h for the 4.3 mM sample of 6^+^. Every sample reached a plateau value within *ca.* 4–5 h after oxidation.

**Fig. 7:**
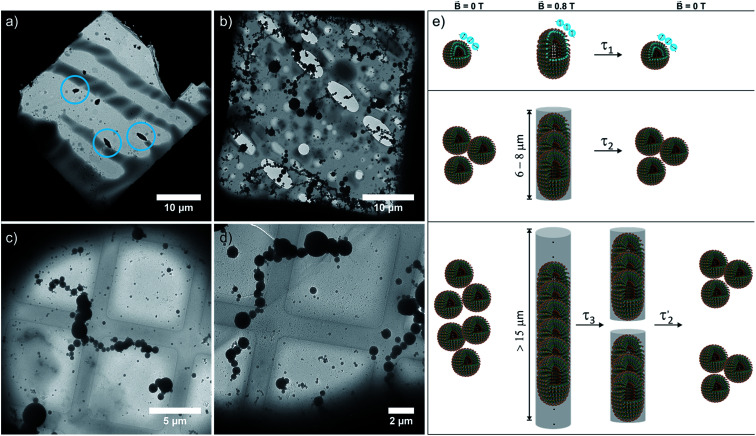
(a) TEM (2.2 mM) and (b)–(d) cryo-TEM images of 3.3 and 4.3 mM samples of 6^+^ collected from our setup after exposing them to the magnetic field (0.8 T) for 5 h. (e) Schematic drawing of the reversible magnetic field-induced deformation of vesicles of paramagnetic amphiphile 6^+^ in a magnetic field and the fusion of individual vesicles into string-of-pearls-like assemblies, and their stepwise disassembly or loss of orientational order after the field is switched off (openings are drawn for better visualization of their inner structures).

**Fig. 8:**
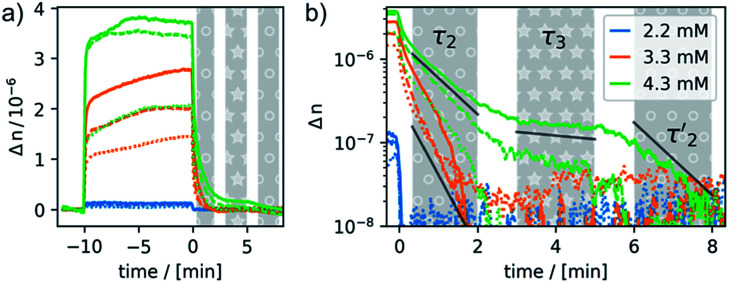
Response and relaxation of magnetically induced birefringence. The field was switched on/off for 10 minutes each. Solid lines: samples aged for 5:10 h; dashed lines: samples aged for 4:30 h; dotted lines: samples aged for 3:10 h after oxidation; the blue, orange or green colors represent the three different concentrations employed in this study. Panel (a) shows the full cycle from switching the field on until full relaxation, and panel (b) shows the relaxation that is observed upon switching the field off. Three distinct timescales can be identified. A fast relaxation occurring within a few seconds is present in all samples. A second relaxation *τ*_2_ of *ca.* 30–60 s is indicated by the shaded gray area with white circles. The birefringence of the sample with a concentration of 4.3 mM does fully not vanish with *τ*_2_ but then decays with a much slower relaxation time *τ*_3_ of 10 min (indicated by the shaded gray area with white star symbols) until *ca.* 5 min (two exponential functions with *τ*_2_ and *τ*_3_ are provided as a guide to the eye). This is followed by a final, faster decay with a relaxation time 
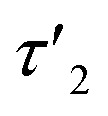
 of 60 s.

The magnetically induced birefringence was measured with modulated laser light under simultaneous DLS monitoring of the diffusion coefficients *D*_‖_ and *D*_⊥_, *i.e.* parallel and orthogonal to the applied magnetic field. The effective hydrodynamic radius as determined from the Stokes–Einstein relation as shown in Fig. 6b and the direction-dependent analysis of the diffusion anisotropy as shown in Fig. S11 of the ESI† agree with the observed changes of the optical birefringence. Since the initial aggregates present a broad size distribution, the appearance and amount of higher aggregates that result from exposing them to the magnetic field do not scale linearly with amphiphile concentration *c* (Δ*n* = 1·10^−7^ at *c* = 2.2 mM, Δ*n* = 3.0·10^−6^ for *c* = 3.3 mM, Δ*n* = 3.8·10^−6^ for *c* = 4.3 mM). The birefringence, which represents the degree of orientation, increases significantly as the amphiphile concentration is changed from 2.2 mM to 3.3 mM, but only moderately so on increasing *c* further to 4.3 mM. Even larger differences are encountered for the diffusion anisotropy, especially for the most concentrated sample (4.3 mM, see Fig. S11 of the ESI†). This indicates that higher aggregates are already present in the 3.3 mM solution. While being sufficiently long to be well-aligned, they still exert only a modest influence on the diffusional behavior. At the even higher concentration of 4.3 mM, the aggregates can grow into very long chains, whose diffusion perpendicular to the axis of orientation is consequently hindered. The latter are detected by their diffusion anisotropy and their persistent orientational order (Fig. 8). These indirectly observed, collective phenomena are in line with TEM and cryo-TEM measurements on samples retrieved after exposing 2.2, 3.3 and 4.3 mM solutions of 6^+^ to the 0.8 T field in our birefringence setup. Representative images as shown in Fig. 7a–d and S13 of the ESI† clearly reveal the formation of deformed aggregates at the lower *c* and of string-of-pearls-like aggregates of slightly deformed vesicles with lengths of up to 30 μm, particularly for 4.3 mM solutions.

Most remarkably, for every pulse of the magnetic field, *i.e.* after switching the magnet on or off, a fast response of the aggregates in solution can be observed (see Fig. 8). Within *ca*. 30–60 s a plateau is reached and only slight changes of the optical birefringence can be observed afterwards. The plateau value of Δ*n* for each pulse scales with the concentrations and average sizes of the spherical aggregates present in solution.

The time-dependent changes of the optical birefringence after the magnetic field is switched off are particularly revealing. The time constant for relaxation is proportional to *r*^3^, where *r* is half the length of the long axis of the particle for both response modes, deformation and orientation. The rotational relaxation time *τ*_rot_ for a diffusing rigid particle is given by

2 *τ*_rot_ ≈ 6π*ηr*^3^/*kT*,

where *η* denotes the viscosity of the medium. By analyzing the relaxation behavior after the field is switched off one can estimate the sizes of the largest aggregates that were formed while the field was switched on.^66,67^

The relaxation behavior of the birefringence Δ*n* is presented in Fig. 8. A fast component with a time constant *τ*_1_ of *ca.* 1 s is present for all samples at every concentration. This component is attributed to the fast relaxation of the population of deformed small aggregates such as the ones shown in Fig. 7a with a size of roughly 1 μm, most probably along the magnetic field axis (*B*_ext_). A second, considerably slower component appears only for the more concentrated samples with *c* = 3.3 mM (*τ*_2_ = 30 s) or 4.3 mM (*τ*_2_ = 60 s). Free rotational diffusion on the timescale of *τ*_2_ corresponds to a structure length of 6–8 μm. The contribution of *τ*_2_ can thus be attributed to the population of mid-sized chain-like arrays of primary aggregates as sketched in the middle right panel of Fig. 7e. The birefringence of the sample at the highest investigated concentration of 4.3 mM displays an additional, even slower decay with a time constant *τ*_3_ of *ca.* 10 min. Such a long time constant corresponds to even more extended structures with lengths of >15 μm (note that, due to their kinked structures, the actual chainlike aggregates are probably even longer, see Fig. 7b–d). Dual DLS measurements of the higher concentrated sample (4.3 mM) reveal that the diffusion coefficient parallel to the external field is *ca.* 1.5 times larger than that measured in a perpendicular direction. This indicates a preferential alignment of the chain-like superstructures parallel to the field (Fig. S14 of the ESI†). The derived diffusion anisotropy ratio of 1.5 is quite large and is fully consistent with the kinked structures of these chains (note that for an ideal, perfectly aligned rod an isotropy factor of 2 would be expected).^68^

5 min after switching the magnetic field off, these extended structures lose their orientational order and probably collapse with a concomitant decay of the birefringence and an associated time constant 
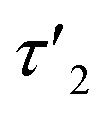
 of 60 s. This final, faster relaxation thus indicates that the larger chains collapse into smaller fragments with a maximum length of *ca.* 8 μm, similar to the length limit obtained at the intermediate concentration. The bottom right image of Fig. 7e provides a sketch of these transformations.^69,70^ The overall behavior of concentrated solutions of 6^+^ is therefore reminiscent of shape-hysteresis materials, as the morphology changes only occur with a delay after an outer perturbation is applied.

## Conclusions and outlook

The novel surfactant FcNMe_2_SO_3_Heptene (6) presented herein offers a unique combination of features that are rooted in utilizing ferrocene as the central building block in conjunction with a zwitterionic sultone headgroup. This renders 6 a multi stimuli-responsive surfactant, whose aggregation behavior can be altered by three different inputs, namely (i) a change of the ionic strength, which increases the solubility of 6 26 fold with a concomitant structure change from intermolecular ion pairs or lamellar structures to first micelles and then vesicles; (ii) reversible oxidation of the ferrocene constituent to paramagnetic 6^+^, which leads to a distinct broadening of the size distribution of these aggregates; and (iii) application of an external magnetic field (0.8 T), which induces a transformation from individual aggregates to chain-like superstructures. Most importantly, we have employed magnetic optical birefringence as a powerful means to monitor the assembly and disassembly of larger, chain-like superstructures *in situ* and in real time. This allowed us to detect a shape-memory-like hysteresis of the structural anisotropy, as orientationally ordered, larger chains persist for *ca.* 5 min after the external magnetic field is switched off. This may open new horizons in dynamic, non-equilibrium self-assembly. At the same time we have demonstrated that magnetic optical birefringence with simultaneous double DLS detection provides a powerful means for the live monitoring of the formation of oriented aggregates of paramagnetic nanoparticles and their subsequent disassembly and the loss of orientational order after the field is switched off. Given our success, we expect that this technique will be of high utility for the future investigation of such systems.

Because this effect and the associated hysteresis are the consequences of a field-induced polarization and a molecular reorientation process, we see similarities to certain aspects from a totally different area, namely the transition from para- to ferromagnetic or from piezo- to ferroelectric materials. As the advent of typical “ferro”-characteristics, such as remanescent behavior, hysteresis patterns, *etc.*, have provided a boost in ensuing properties and applications, we are confident this can also be the case for surfactants with ferro-self-assembly features.

## Methods

### General

NMR experiments were carried out on a Varian Unity Inova 400, a Bruker Avance III DRX 400, or a Bruker Avance Neo 800 MHz spectrometer. ^1^H and ^13^C NMR spectra were referenced to the respective solvent signal. 2D-NMR experiments were used to unequivocally assign the NMR resonances. The numbering of the nuclei is provided in Fig. S1 of the ESI.† ESIMS data were acquired on a Bruker microTOF focus II system. Cyclic voltammetry was performed in a one-compartment cell with 5–7 mL of deionized water as the solvent and KNO_3_ (0.1 M) as the supporting electrolyte. A gold electrode (*∅* = 1.1 mm, BASI) was used as the working electrode. A computer controlled BASi EPSILON potentiostat was used for recording the voltammograms. An Ag/AgCl (3 M KCl) reference electrode in water was used in combination with a platinum wire auxiliary electrode. IR/NIR spectra were recorded on a FT-IR Bruker Tensor II instrument. UV/Vis spectroscopy was performed on a diode-array unit TIDAS manufactured by J&M ANALYTIK. ATR-IR spectra were measured on a PerkinElmer 100 spectrometer equipped with an ATR unit. DLS measurements were performed on a Malvern Zen5600 instrument. The liquid crystal picture was obtiained with an Olympus CX41 light microscope. For surface tension measurements, a Krüss K100 instrument was used. Additional surface tension measurements were performed by the capillary method. SAXS measurements were acquired on a Bruker Nanostar system equipped with a pinhole collimator and a Cu K_α_ radiation source. Powder X-ray diffraction measurements (PXRD) were performed with a Bruker AXS D8 Avance diffractometer using Cu-K_α_ radiation and a lynxeye detector. Geometry optimization and orbital calculations were performed using DFT with the Gaussian16 package for *ab initio* electronic structure calculation using the pbe1pbe/def2-TZVP level of theory.^71–75^ The GaussSum program package was used to analyze the results, while the visualization of the results was performed with the Avogadro program package.^76,77^ Graphical representations of molecular orbitals were generated with the help of GNU parallel and plotted using the vmd program package in combination with POV-Ray.^78–80^ Electron microscopy was performed on a Zeiss Libra 120 TEM instrument and a JEOL JEM 2200FS with accelerating voltages of 120 and 200 kV, respectively. Both microscopes were equipped with in column energy filters. For cryo-TEM measurements the samples were prepared using a Grid-Plunger Leica EM GP. 3 μL of the dispersion were dropped onto both sides of a Quantifoil holey carbon film grid. The excess liquid was removed with a filter paper and the resulting thin water films were vitrified by rapidly immersing (plunging) into liquid ethane. The specimens were then inserted into a cryo-transfer sample holder Gatan 914 and transferred to the TEM instrument. X-Ray diffraction analysis on single crystals of 6 grown from acetonitrile was performed on a STOE IPDS-II diffractometer equipped with a graphite-monochromated Mo K_α_ radiation source (*λ* = 0.71073 Å) and an image plate detection system at 100.15 K. Using Olex2, the structures were solved with the SIR2004 structure solution program using direct methods or the ShelXT structure solution program using Intrinsic Phasing and refined with the ShelXL refinement package using Least Squares minimization.^81–84^ Hydrogen atoms were introduced at their calculated positions. Optical *in situ* spectroscopy was performed as follows: the sample is placed in an electromagnet and illuminated with a laser (HeNe 632 nm, 10 mW). The birefringence is detected from the transmitted beam. Enhanced detection sensitivity is achieved with the use of a photoelastic modulator (PEM II/FS84 from Hinds Instruments), two Glan–Thompson polarizers (aligned at ±45° with respect to the magnetic field) and a lock-in amplifier (Stanford ResearchSR830-DSP). The value of birefringence is measured by compensation of the signal with a pockels cell.^85,86^ The two DLS detection optics are aligned at a scattering angle of 9°, one with a horizontal and the other with a vertical scattering plane. The scattered light is collected using mono-mode fibers and detected with single photon detectors (ALV SO-SIPD) that are connected to a hardware correlator (Flex02-12D/C from http://correlator.com). The intensity autocorrelation functions are analyzed separately with a cumulant method. The diffusion anisotropy is calculated directly from the ratio of the first cumulants. The effective hydrodynamic radius is calculated from the average diffusion coefficient, using the Stokes–Einstein relation. The polydispersity is estimated from the second cumulant.^87^

### Synthesis and characterization

The reactions were performed using standard Schlenk techniques under a N_2_ atmosphere. Solvents were dried according to standard procedures and stored under an argon atmosphere. Water was deionized using a Millipore Milli-Q. C_6_D_6_, CD_2_Cl_2_, CDCl_3_, D_2_O and MeOH-*d*_4_ were supplied by Eurisotop. Starting materials for syntheses were purchased from commercial sources unless stated otherwise. Hexyltriphenylphosphonium bromide (Ph_3_PHexyl^+^Br^−^),^88^ 1,1′-dibromoferrocene (FcBr_2_, (2)),^51^ and 1-formyl-1′-bromoferrocene (FcBrCHO, (3))^54^ were prepared according to the literature. Detailed procedures are provided in the ESI.† The atom numbering pertinent to the NMR discussion is provided in the ESI† together with the corresponding NMR spectra.

### 1-(*Z*)-Heptenyl-1′-bromoferrocene (FcBrHeptene, 4)

BrPPh_3_Hex (3.25 g, 7.61 mmol, 1 equiv.) was dissolved in 60 mL of THF. After the addition of KO^t^Bu (0.85 g, 7.61 mmol, 1 equiv.) to the turbid solution, a colour change to red was observed. The solution was stirred for 1 h. A solution of FcBrCHO (2.23 g, 7.61 mmol, 1 equiv.) in 20 mL of THF was added dropwise over a period of 20 min. The solution was stirred overnight. 40 mL of *n*-pentane were added, and the precipitate was filtered off. The solvent was removed *in vacuo* and the crude product was purified by column chromatography (50% EE/PE) yielding FcBrHeptene (2.52 g, 7.00 mmol, 92%) as a brown oil. FcBrHeptene was obtained selectively in (*Z*)-configuration. ^1^H NMR (400 MHz, CDCl_3_) *δ* 6.06 (dt, ^3^*J*_HH,cis_ = 11.3 Hz, ^4^*J*_HH_ = 1.8 Hz, 1H, CpC-H), 5.55 (dt, ^3^*J*_HH,cis_ = 11.3 Hz, ^3^*J*_HH_ = 7.2 Hz, 1H, C=CH), 4.32 (vt, ^3^*J*_HH_ = 1.9 Hz, 2H, Cp-H), 4.30 (vt, ^3^*J*_HH_ = 1.9 Hz, 2H, Cp-H), 4.26 (vt, ^3^*J*_HH_ = 1.9 Hz, 2H, Cp-H), 4.05 (vt, ^3^*J*_HH_ = 1.9 Hz, 2H, Cp-H), 2.25–2.22 (m, 2H, CH_2_), 1.51–1.42 (m, 2H, CH_2_), 1.39–1.32 (m, 4H, CH_2_), 0.92 (t, ^3^*J*_HH_ = 7.2 Hz, CH_3_).

### 1-(*Z*)-Heptenyl-1′-dimethylaminomethylferrocene (FcNMe_2_Heptene, 5)

FcBrHeptene (1.14 g, 3.16 mmol, 1 equiv.) was dissolved in 50 mL of THF and the solution was cooled to −78 °C. Then, 2.0 mL of a 1.6 M solution of *n*-BuLi in hexane (3.16 mmol, 1 equiv.) were added dropwise over a period of 5 min. The solution was stirred at −78 °C for another 10 min. Eschenmoser's salt (585 mg, 3.16 mmol, 1 equiv.) was added and the temperature was kept at −78 °C for one hour. Then, 20 mL of distilled water and 40 mL of ethyl acetate were added. The phases were separated, and the aqueous phase was extracted twice with ethyl acetate. The combined organic layers were washed with brine and dried over MgSO_4_. The solvent was removed *in vacuo* and the crude product was purified by column chromatography (5% NEt_3_/PE) yielding 400 mg of FcNMe_2_Heptene (1.18 mmol, 37%) as a brown oil. ^1^H NMR (400 MHz, CDCl_3_) *δ* 6.02 (d, ^3^*J*_HH,cis_ = 11.5 Hz, 1H, CpC-H), 5.51 (dt, ^3^*J*_HH,cis_ = 11.5 Hz, ^3^*J*_HH_ = 7.2 Hz, 1H, C=CH), 4.28–4.24 (m, 2H, Cp-H), 4.18–4.15 (m, 2H, Cp-H), 4.11–4.06 (m, 4H, Cp-H), 3.25 (s, 2H, NCH_2_), 2.26–2.24 (m, 2H, CH_2_), 2.18 (s, 6H, NCH_3_), 1.52–1.42 (m, 2H, CH_2_), 1.41–1.34 (m, 2H, CH_2_), 0.94 (t, ^3^*J*_HH_ = 7.0 Hz, CH_3_).

### 1-(*Z*)-Heptenyl-1′-dimethylammoniummethyl-(3-sulfopropyl)-ferrocene (FcNMe_2_SO_3_Heptene, 6)

A solution of FcNMe_2_Heptene (400 mg, 1.18 mmol, 1 equiv.) and 1,3-propane sultone (171 mg, 1.40 mmol, 1.2 equiv.) in 40 mL of acetonitrile was heated to reflux for 15 h. After cooling to room temperature, the solvent was removed *in vacuo*. The orange precipitate was washed four times with toluene (3 mL) yielding 380 mg of FcNMe_2_SO_3_Heptene (0.82 mmol, 70%). ^1^H NMR (400 MHz, MeOD) *δ* 6.10 (dt, ^3^*J*_HH,cis_ = 11.4 Hz, ^4^*J*_HH_ = 1.7 Hz, 1H, H-12), 5.61 (dt, ^3^*J*_HH,cis_ = 11.4 Hz, ^3^*J*_HH_ = 7.3 Hz, 1H, H-13), 4.48 (vt, ^3^*J*_HH_ = 1.9 Hz, 2H, H-7), 4.45 (vt, ^3^*J*_HH_ = 1.9 Hz, 2H, H-10), 4.38 (vt, ^3^*J*_HH_ = 1.9 Hz, 2H, H-8), 4.37 (s, 2H, H-5), 4.34 (vt, ^3^*J*_HH_ = 1.9 Hz, 2H, H-9), 3.45–3.39 (m, 2H, H-3), 2.96 (s, 6H, H-4), 2.88 (t, ^3^*J*_HH_ = 6.9 Hz, 2H, H-1), 2.31–2.15 (m, 4H, H-2, H-14), 1.54–1.45 (m, 2H, H-15), 1.43–1.35 (m, 4H, H-16, H-17), 0.95 (t, ^3^*J*_HH_ = 7.1 Hz, 3H, H-18). ^13^C NMR (151 MHz, CDCl_3_) *δ* 131.67 (s, C-13), 124.53 (s, C-12), 84.07 (s, C-11), 72.86 (s, C-7), 72.35 (s, C-9), 71.97 (s, C-6), 70.36 (s, C-10), 70.15 (s, C-8), 65.68 (s, C-5), 63.21 (s, C-3), 49.55 (s, C-4), 48.07 (s, C-1), 31.80 (s, C-16), 29.53 (s, C-15), 29.19 (s, C-14), 22.74 (s, C-17), 19.73 (s, C-2), 14.25 (s, C-18). ESIMS: [g mol^−1^]: (4M + Na + H)^+^ = (C_92_H_141_Fe_4_N_4_O_12_S_4_Na)^+^ calc.: 1868.67, found: 1868.67; (4M + Na)^+^ = (C_92_H_140_Fe_4_N_4_O_12_S_4_Na)^+^ calc.: 1867.66, found: 1867.66; (3M + Na)^+^ = (C_69_H_105_Fe_3_N_3_O_9_S_3_Na)^+^ calc.: 1406.50, found: 1406.50; (2M + Na)^+^ = (C_46_H_70_Fe_2_N_2_O_6_S_2_Na)^+^ calc.: 945.33, found: 945.33; (2M + H)^+^ = (C_46_H_71_Fe_2_N_2_O_6_S_2_)^+^ calc.: 923.34, found: 923.34; (M + K)^+^ = (C_23_H_35_FeNO_3_SK)^+^ calc.: 500.13, found: 500.13; (M + Na)^+^ = (C_23_H_35_FeNO_3_SNa)^+^ calc.: 484.16, found: 484.15; (M + H)^+^ = (C_23_H_36_FeNO_3_S)^+^ calc.: 462.18, found: 462.17; (M)^+^ = (C_23_H_35_FeNO_3_S)^+^ calc.: 461.17, found: 461.17; (M − NMe_2_SO_3_)^+^ = (C_18_H_23_Fe)^+^ calc.: 295.11, found: 295.11. IR (powder): 3067, 3037, 2955, 2918, 2853, 1638. UV-Vis (MeCN): = 225 M^−1^ cm^−1^.

## Conflicts of interest

The authors declare no competing financial interests.

## Supplementary Material

SC-012-D0SC05249C-s001

SC-012-D0SC05249C-s002
