# Supplementary material for: Ferro-self-assembly: magnetic and electrochemical adaptation of a multiresponsive zwitterionic metalloamphiphile showing a shape-hysteresis effect
Source: Chem Sci. 2020 Nov 3;12(1):270–81. doi: 10.1039/d0sc05249c (PMC8178951; doi:10.1039/d0sc05249c)
Supplement: SC-012-D0SC05249C-s001 [file SC-012-D0SC05249C-s001.pdf]

Electronic Supplementary Material (ESI) for Chemical Science.
This journal is © The Royal Society of Chemistry 2020

Ferro-Self-Assembly: Magnetic and Electrochemical Adaptation of a Multiresponsive Zwitterionic Metalloamphiphile Showing a Shape-Hysteresis Effect

Stefan Bitter[a], Moritz Schlötter[a], Markus Schilling[a], Marina Krumova[a], Sebastian Polarz[ab]*, and Rainer F. Winter[a]*

[a]Department of Chemistry, University of Konstanz, Universitätsstrasse 10, 78457 Konstanz (Germany)

[b]Institute of Inorganic Chemistry, Leibniz-University Hannover, Callinstrasse 9, 30167 Hannover (Germany)

Keywords: Ferrocene, Surfactant, Amphiphile, Self-Assembly, Multi-Stimuli Responsive, Redox-Responsive, Magneto-Responsive, Birefringence, Shape-Hysteresis Effect

E-mail: sebastian.polarz@aca.uni-hannover.de
rainer.winter@uni-konstanz.de

Supporting Information

Supporting Information **1**

Synthesis and Characterization **2**

Supporting Figures **3**

Fig S1: Synthesis and Characterization of $FcNMe_2SO_3$Heptene **6**. 3

Fig. S2: DFT calculated frontier orbitals of **6**. 6

Fig. S3: Intramolecular ionic pairs. 7

Fig. S4: Crystal structure of **6** on crystals grown from acetonitrile. 8

Fig. S5: SAXS and PXRD measurements of **6**. 10

Fig. S6: DOSY measurements of **6**. 12

Fig. S7: Zeta Potential measurements of **6**. 13

Fig. S8: Typical surfactant properties of **6**. 14

Fig. S9: EELS of **6**. 16

Fig. S10: Oxidation of **6** to **6^+** and characterization of **6^+**. 17

Fig. S11: Surfactant properties of **6^+**. 20

Fig. S12: EELS of **6^+**. 22

Fig. S13: Additional Cryo-TEM images of **6^+** after exposure to the magnetic field 23

Fig. S14: Diffusion anisotropy factor of **6^+** obtained from DLS orthogonal and parallel to the external magnetic field 24

References **25**

Synthesis and Characterization

Hexyltriphenylphosphonium bromide ($Ph_3PHexyl^+Br^-$). 1-Bromo-hexane (6.0 mL, 42.4 mmol, 1 equiv) and PPh_3 (11.55 g, 44.5 mmol, 1.05 equiv) were dissolved in 70 mL of toluene. The solution was heated to reflux for 15 h. After cooling to room temperature, a white precipitate was obtained. It was filtered, washed three times with 20 mL of pentane, and dried *in vacuo*. $Ph_3PHexyl^+Br^-$ (8.44 g, 19.08 mmol, 45%) was obtained as a colourless solid. ^1H NMR (400 MHz, $CDCl_3$): δ 7.91-7.83 (m, 6H, Ph-H), 7.81-7.74 (m, 3H, Ph-H), 7.73-7.66 (m, 6H, Ph-H), 3.91-3.82 (m, 2H, PCH_2), 1.60-1.67 (m, 4H, CH_2), 1.26-1.18 (m, 4H, CH_2), 0.82 (t, $^3J_{HH}$ = 6.9 Hz, 3H, CH_3).

1,1'-dibromoferrocene ($FcBr_2$, 2). A solution of ferrocene (10 g, 53.75 mmol, 1 eq.), *n*-hexane (400 mL) and TMEDA (19 mL, 125.24 mmol, 2.33 eq.) was stirred in a dried 1 L schlenk flask and cooled to 0 °C. Then 1.6 M *n*-BuLi in hexane (72 mL, 125.24 mmol, 2.33 eq.) was added dropwise and the suspension was raised to room temperature over night. The orange precipitate was filtered, re-suspended in diethyl ether (350 mL) and cooled to -78 °C and a solution of 13.5 mL tetrabromoethane (TBE) (115.56 mmol, 2.15 eq.) in 80 mL diethyl ether was added dropwise. The solution was raised to ambient temperature over night. The dark red solution was decanted and quenched with 100 mL of water. After solvent removal the dark orange solid was dissolved in 300 mL hexane and filtered trough celite and then washed subsequently with sat. *aq*. $FeCl_3$ (*ca*. 3 × 100 mL). The organic phase was extracted with water, dried over $MgSO_4$ and the solvent was removed *in vacuo*. Pure orange crystalline $FcBr_2$ was obtained after recrystallization from MeOH in 59 % yield (10.89 g, 31.67 mmol). ^1H NMR (400 MHz, $CDCl_3$): δ 4.42 (vt, $^3J_{HH}$ = 1.9 Hz, 4H, Cp-H), 4.17 (vt, $^3J_{HH}$ = 1.9 Hz, 4H, Cp-H).

1-formyl-1'-bromoferrocene (FcBrCHO, 3). $FcBr_2$ (9.32 g, 27.11 mmol, 1 equiv) was dissolved in 120 mL THF and the solution was cooled to -78 °C. Then, 16.9 mL of a 1.6 M solution of *n*-BuLi in hexane (27.11 mmol, 1 equiv) were added dropwise over a period of 20 min. The red solution was stirred at -78 °C for further 30 min. DMF (3.3 mL, 43.4 mmol, 1.6 equiv) was added dropwise over a period of 10 min. The mixture was stirred for further 30 min at -78 °C and for 2 h at room temperature. The reaction was quenched with 20 mL of 1 M HCl and 20 mL of saturated NaCl solution. The phases were separated, and the aqueous phase was extracted twice with 15 mL of diethyl ether. The combined organic phases were dried over $MgSO_4$ and the solvent was removed *in vacuo*. The crude product was purified by column chromatography (1-9% EE/PE) yielding FcBrCHO (7.40 g, 25.26 mmol, 93%) as dark red needles. ^1H NMR (400 MHz, $CDCl_3$) δ 9.99 (s, 1H, CHO), 4.84 (vt, $^3J_{HH}$ = 1.9 Hz, 2H, Cp-H), 4.63 (vt, $^3J_{HH}$ = 1.9 Hz, 2H, Cp-H), 4.52 (vt, $^3J_{HH}$ = 1.9 Hz, 2H, Cp-H), 4.21 (vt, $^3J_{HH}$ = 1.9 Hz, 2H, Cp-H).

Supporting Figures

Fig S1: Synthesis and Characterization of FcNMe$_2$SO$_3$Heptene **6.**

(a) ^{1}H NMR of **6**.

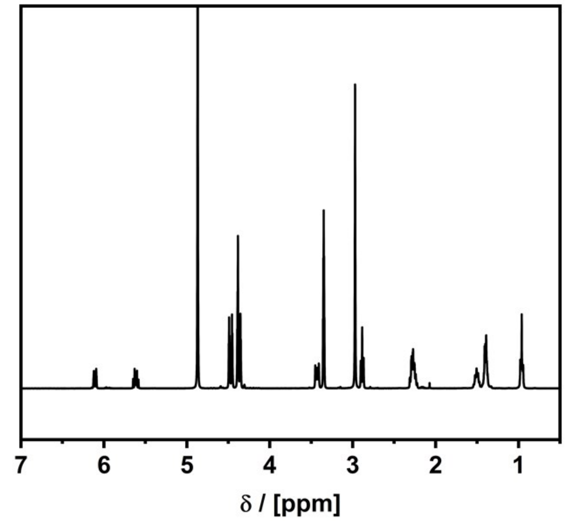

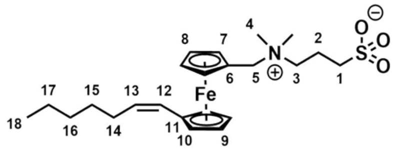

FcNMe$_2$SO$_3$Heptene (6/6$^+$)

^{1}H NMR (400 MHz, MeOD) δ [ppm]: 6.10 (dt, $^{3}J_{HH,cis}$ = 11.4 Hz, $^{4}J_{HH}$ = 1.7 Hz, 1H, **H-12**), 5.61 (dt, $^{3}J_{HH,cis}$ = 11.4 Hz, $^{3}J_{HH}$ = 7.3 Hz, 1H, **H-13**), 4.48 (vt, $^{3}J_{HH}$ = 1.9 Hz, 2H, **H-7**), 4.45 (vt, $^{3}J_{HH}$ = 1.9 Hz, 2H, **H-10**), 4.38 (vt, $^{3}J_{HH}$ = 1.9 Hz, 2H, **H-8**), 4.37 (s, 2H, **H-5**), 4.34 (vt, $^{3}J_{HH}$ = 1.9 Hz, 2H, **H-9**), 3.45-3.39 (m, 2H, **H-3**), 2.96 (s, 6H, **H-4**), 2.88 (t, $^{3}J_{HH}$ = 6.9 Hz, 2H, **H-1**), 2.31-2.15 (m, 4H, **H-2**, **H-14**), 1.54-1.45 (m, 2H, **H-15**), 1.43-1.35 (m, 4H, **H-16**, **H-17**), 0.95 (t, $^{3}J_{HH}$ = 7.1 Hz, 3H, **H-18**).

(b) ^{13}C{^{1}H} NMR of **6**.

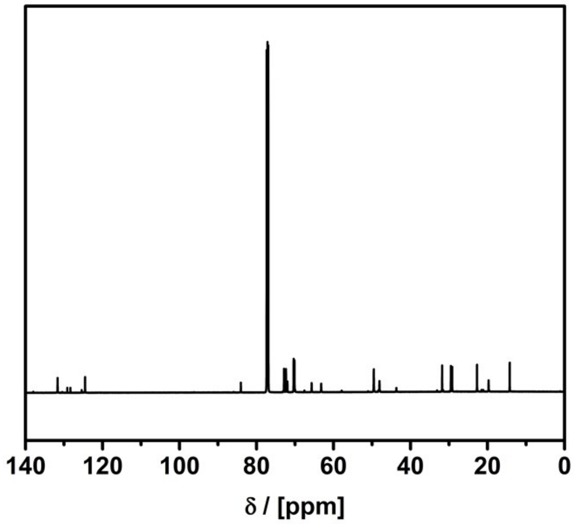

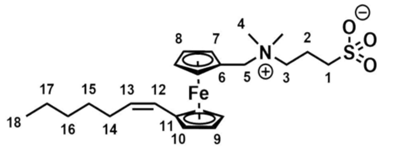

FcNMe$_2$SO$_3$Heptene (6/6$^+$)

^{13}C NMR (151 MHz, CDCl$_3$) δ [ppm]: 131.67 (s, **C-13**), 124.53 (s, **C-12**), 84.07 (s, **C-11**), 72.86 (s, **C-7**), 72.35 (s, **C-9**), 71.97 (s, **C-6**), 70.36 (s, **C-10**), 70.15 (s, **C-8**), 65.68 (s, **C-5**), 63.21 (s, **C-3**), 49.55 (s, **C-4**), 48.07 (s, **C-1**), 31.80 (s, **C-16**), 29.53 (s, **C-15**), 29.19 (s, **C-14**), 22.74 (s, **C-17**), 19.73 (s, **C-2**), 14.25 (s, **C-18**).

(c) ESI MS of **6** in positive mode.

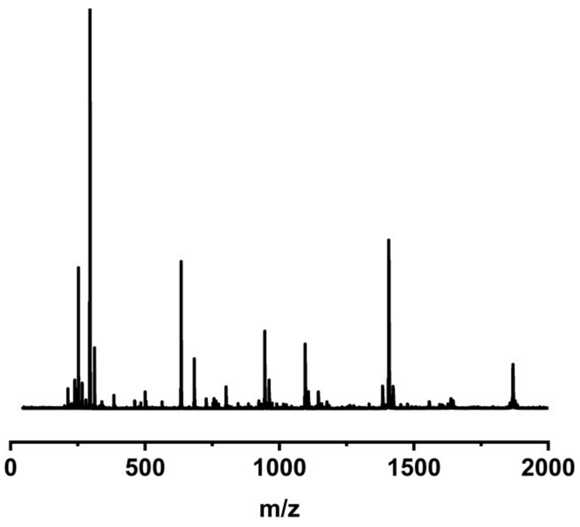

(d) Zoom-in versions of ESIMS of (**6**) in positive mode. Left: $(M+H)^+ = (C_{23}H_{36}FeNO_3S)^+$ calc.: 462.18, found: 462.17; $(M)^+ = (C_{23}H_{35}FeNO_3S)^+$ calc.: 461.17, found: 461.17; Right: $(2M+Na)^+ = (C_{46}H_{70}Fe_2N_2O_6S_2Na)^+$ calc.: 945.33, found: 945.33.

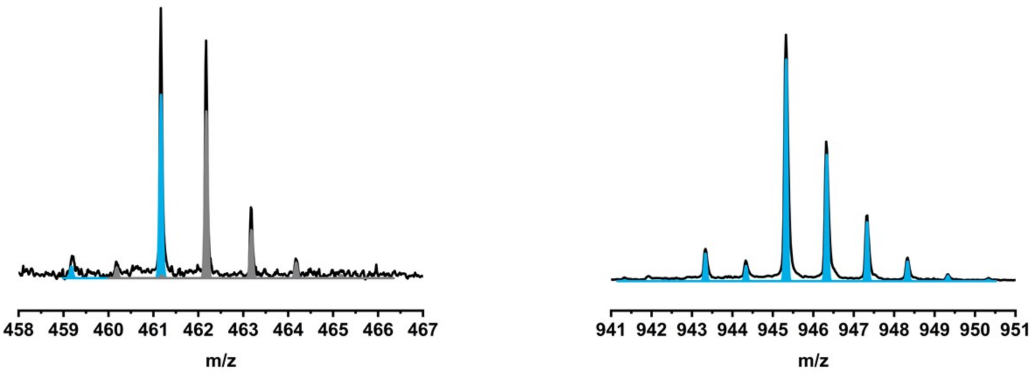

ESI-MS [$gmol^{-1}$]: $(4M+Na+H)^+ = (C_{92}H_{141}Fe_4N_4O_{12}S_4Na)^+$ calc.: 1868.67, found: 1868.67; $(4M+Na)^+ = (C_{92}H_{140}Fe_4N_4O_{12}S_4Na)^+$ calc.: 1867.66, found: 1867.66; $(3M+Na)^+ = (C_{69}H_{105}Fe_3N_3O_9S_3Na)^+$ calc.: 1406.50, found: 1406.50; $(2M+Na)^+ = (C_{46}H_{70}Fe_2N_2O_6S_2Na)^+$ calc.: 945.33, found: 945.33; $(2M+H)^+ = (C_{46}H_{71}Fe_2N_2O_6S_2)^+$ calc.: 923.34, found: 923.34; $(M+K)^+ = (C_{23}H_{35}FeNO_3SK)^+$ calc.: 500.13, found: 500.13; $(M+Na)^+ = (C_{23}H_{35}FeNO_3SNa)^+$ calc.: 484.16, found: 484.15; $(M+H)^+ = (C_{23}H_{36}FeNO_3S)^+$ calc.: 462.18, found: 462.17; $(M)^+ = (C_{23}H_{35}FeNO_3S)^+$ calc.: 461.17, found: 461.17; $(M-NMe_2SO_3)^+ = (C_{18}H_{23}Fe)^+$ calc.: 295.11, found: 295.11.

(e) Infrared spectrum of **6**.

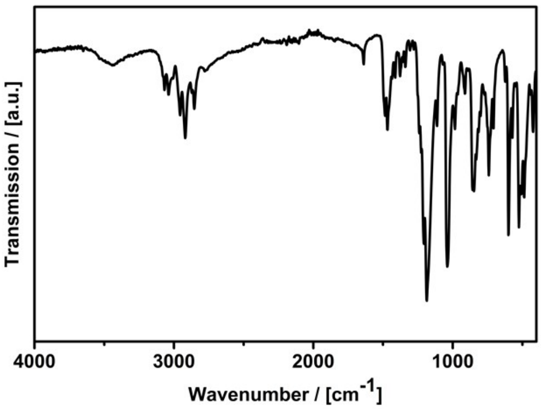

IR (ATR, [cm^{-1}]): 3067, 3037 (**=C-H**); 2955, 2918, 2853 (**-C-H**); 1638 (**C=C**); 1185 (ν_{as} **SO_3^-**), 1037 (ν_s **SO_3^-**).

(f) UV/Vis spectrum of **6** recorded in acetonitrile.

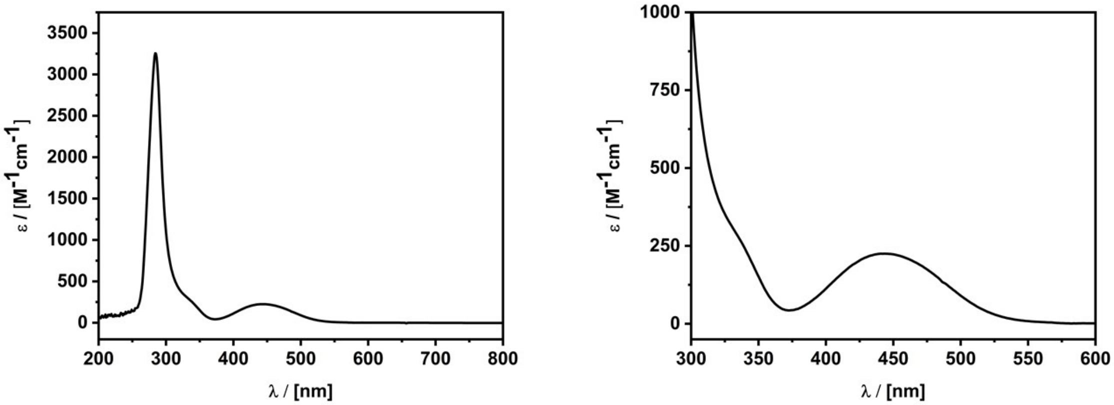

UV-Vis (MeCN): = 225 $M^{-1}cm^{-1}$.

(g) Photographic image of **6**.

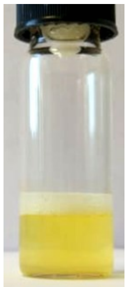

Fig. S2: DFT calculated frontier orbitals of **6**.

(a) Geometry optimization and orbital calculations were performed using DFT with the Gaussian16 program package for ab initio electronic structure calculation using the pbe1pbe/def2-TZVP level of theory. Left: HOMO; Right: LUMO.

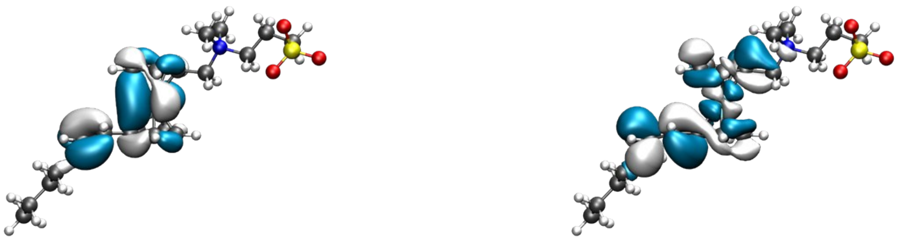

Fig. S3: Intramolecular ionic pairs.

(a) NOESY spectrum of compound **6** performed in $CDCl_3$.

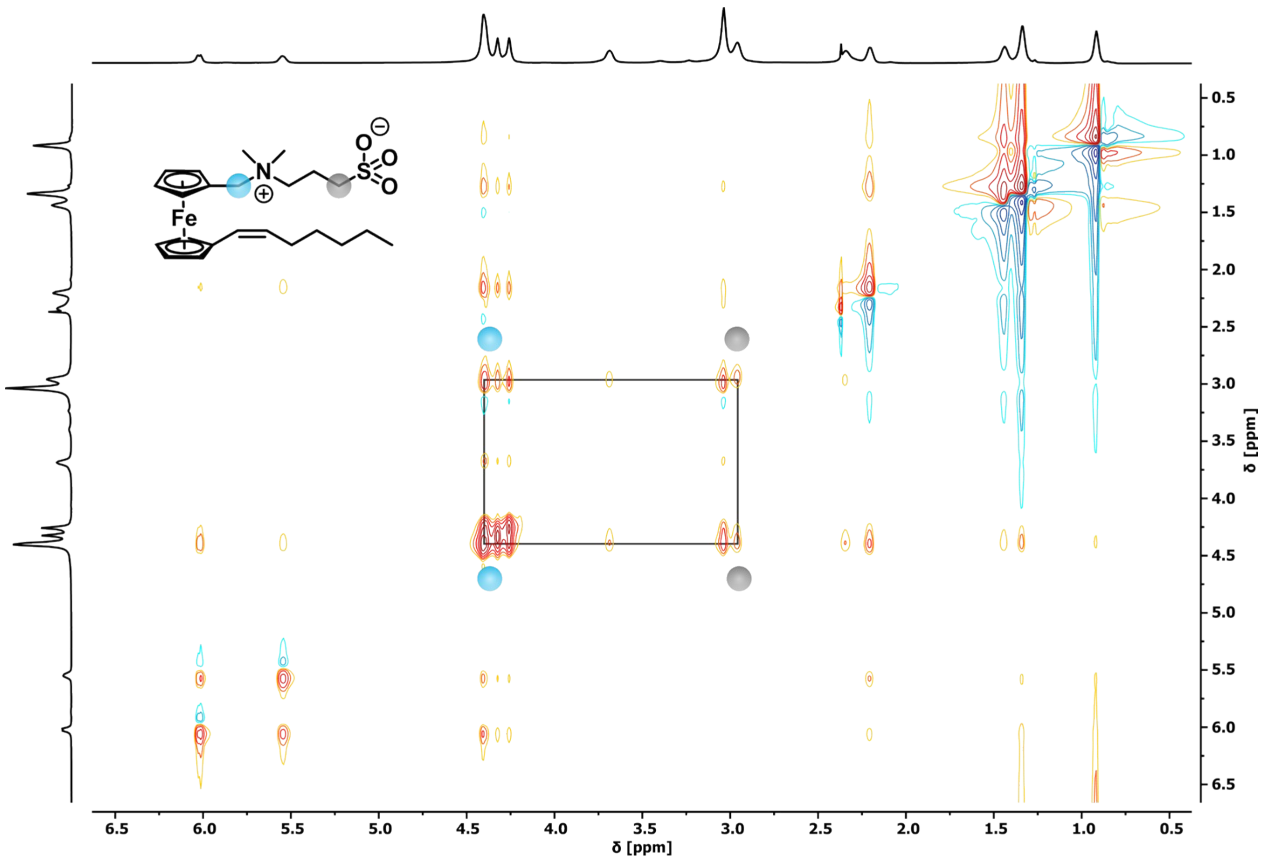

The resonance signal at δ = 4.41 ppm corresponds to the CH_2-group between the Cp and the ammonium. These protons show a strong through-space coupling with the CH_2-group next to the sulfonate (δ = 2.95 ppm). The sulfonate forms an intra- or intermolecular ionic pair with the ammonium.

Fig. S4: Crystal structure of **6** on crystals grown from acetonitrile.

(a) Crystal structure of **6** crystallized from acetonitrile. C dark grey, N turquoise, Fe orange, S yellow, O red; H atoms are omitted; grey lines denote the unit cell.

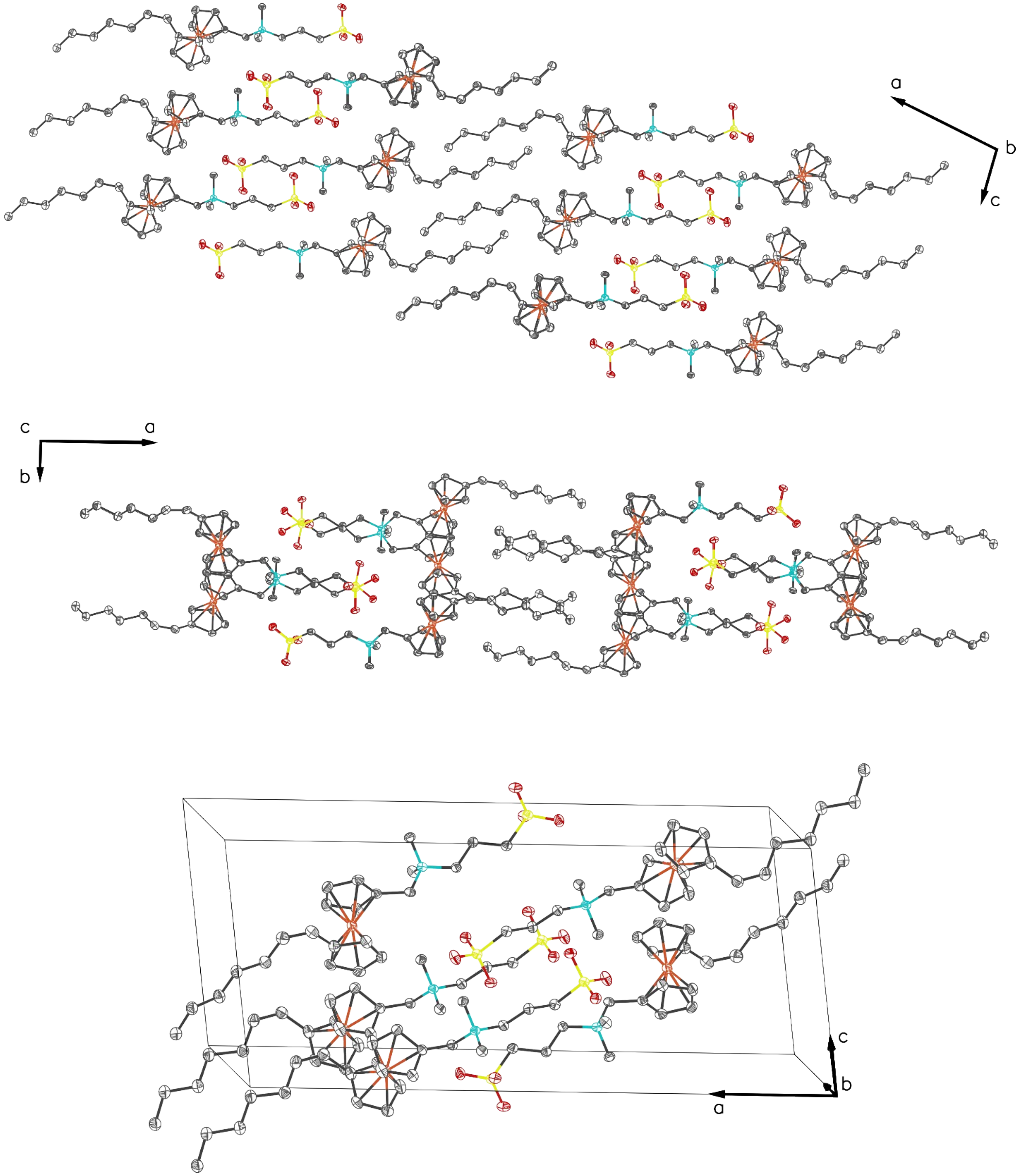

Table 1 Crystal data and structure refinement for FcNMe2SO3Heptene.

Identification code	FcNMe2SO3Heptene
Empirical formula	$C_{23}H_{35}FeNO_3S$
Formula weight	461.43
Temperature/K	100
Crystal system	monoclinic
Space group	$P2_1/c$
a/Å	26.9077(6)
b/Å	7.4341(3)
c/Å	11.4663(16)
α/°	90
β/°	100.496(4)
γ/°	90
Volume/Å^3	2255.3(3)
Z	4
ρ_{calc}g/cm^3	1.359
μ/mm^{-1}	0.785
F(000)	984.0
Crystal size/mm^3	0.5 × 0.4 × 0.1, orange plate
Radiation	MoKα (λ = 0.71073)
2Θ range for data collection/°	4.618 to 55.996
Index ranges	-35 ≤ h ≤ 34, -9 ≤ k ≤ 9, -13 ≤ l ≤ 15
Reflections collected	20514
Independent reflections	5432 [R_{int} = 0.0438, R_{sigma} = 0.0317]
Data/restraints/parameters	5432/0/266
Goodness-of-fit on F^2	1.082
Final R indexes [I>=2σ (I)]	R_1 = 0.0463, wR_2 = 0.1012
Final R indexes [all data]	R_1 = 0.0682, wR_2 = 0.1147
Largest diff. peak/hole / e Å^{-3}	0.35/-0.53

Fig. S5: SAXS and PXRD measurements of **6**.

(a) SAXS measurements of **6** performed in pure water (left) and 0.01M aqueous KPF_6 (right).

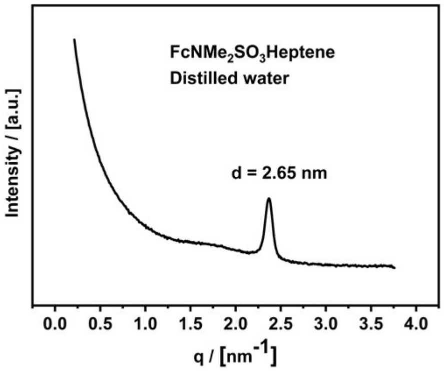

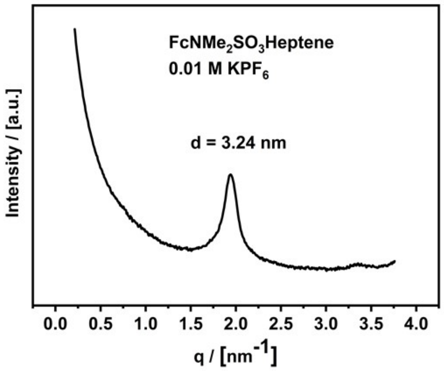

(b) PXRD measurements of **6** performed in pure water (left) and 0.01M aqueous KPF_6 (right).

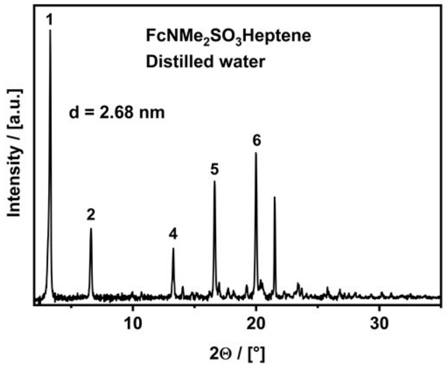

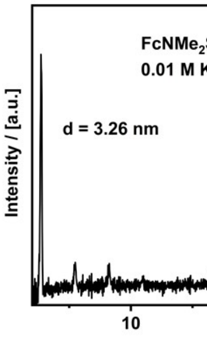

	PXRD 2Theta [°]	PXRD d [nm]	SAXS d [nm]
1	3.3	2.68	2.65
2	6.61	1.34	
4	13.29	0.67	
5	16.64	0.53	
6	19.99	0.44	

	PXRD 2Theta [°]	PXRD d [nm]	SAXS d [nm]
1	2.71	3.26	3.24
2	5.49	1.61	
3	8.25	1.07	

(c) Calculated PXRD pattern of **6** (crystallized from acetonitrile) compared to PXRD pattern of **6** obtained in pure water.

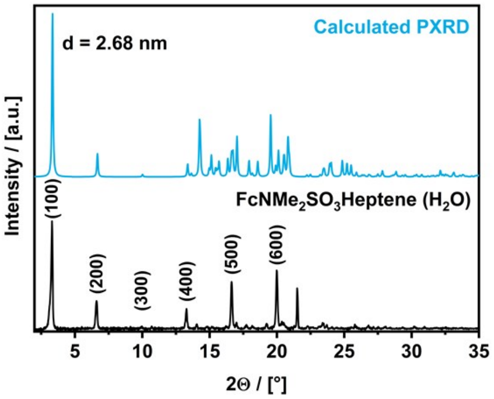

Fig. S6: DOSY measurements of **6**.

(a) Attenuated intensity of **6** as a function of the applied gradient strength. A non-linear Gaussian regression fit was used. The result fulfills the diffusion theory and the *Stejskal-Tanner* equation.[1-3]

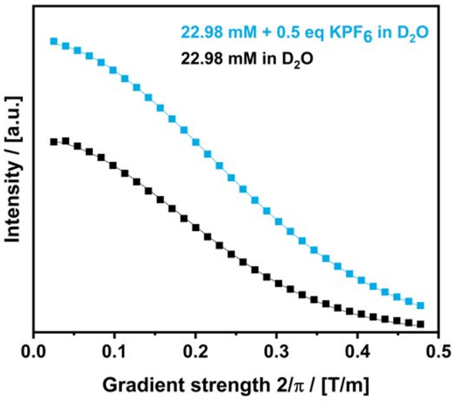

Stejskal-Tanner equation (non-linear Gaussian):

Calculated diffusion coefficient

	[m²/s]
22.98 mM D_2O	
22.98 mM + 0.5 eq KPF_6 in D_2O	

Fig. S7: Zeta Potential measurements of **6**.

(a) Cation influence on the Zeta Potential (0.01 M aqueous electrolyte solution).

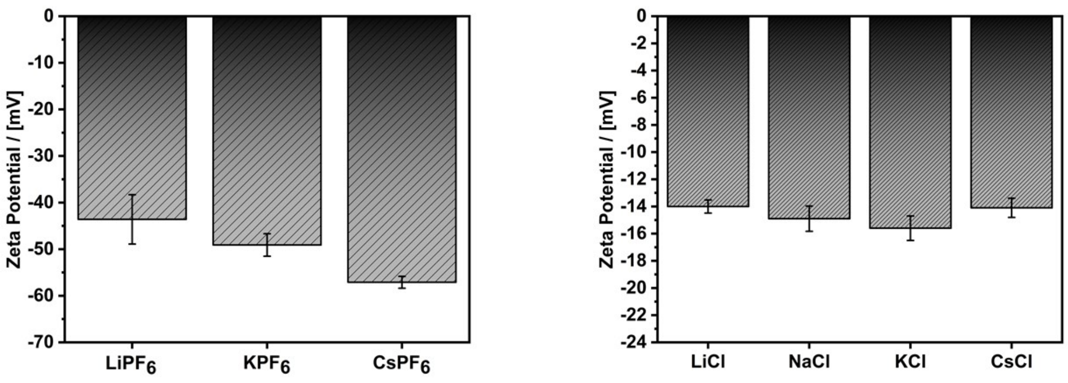

(b) Anion influence on the Zeta Potential (0.01 M aqueous electrolyte solution).

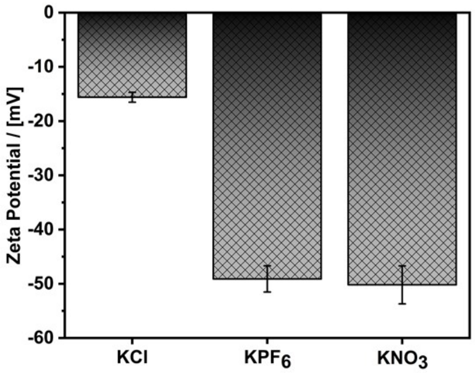

(c) Electrolyte concentration influence on the Zeta Potential (0.01 and 0.1 M electrolyte solution).

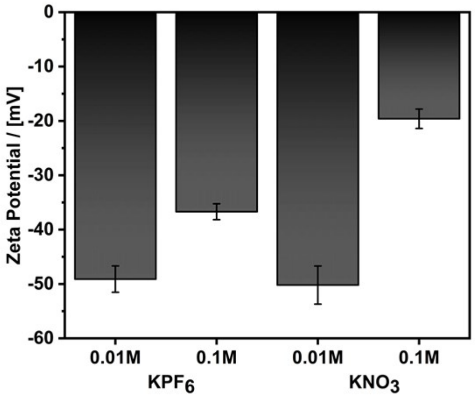

Fig. S8: Typical surfactant properties of **6**.

(a) DLS data of **6** measured in 0.1M KNO_3 directly after dilution (t=0d, black) and after 5 days (t=5d, grey).

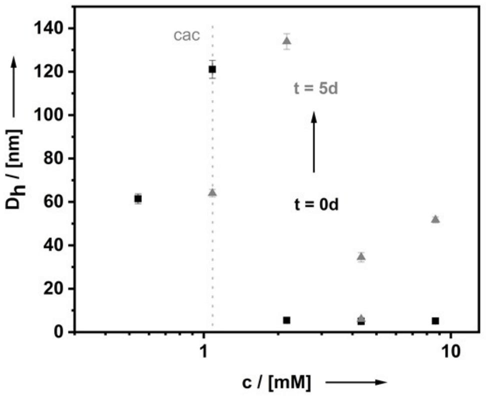

(b) DLS data of **6** measured in 0.01M KPF_6 directly after dilution (t=0d, black) and after 5 days (t=5d, grey).

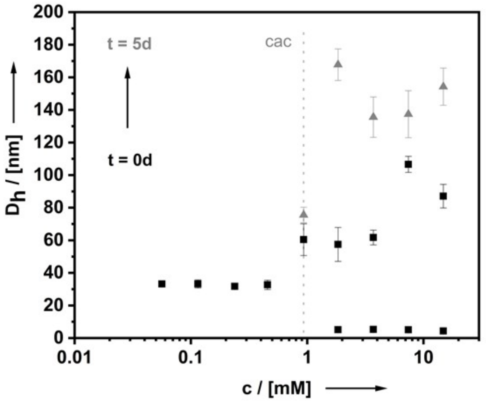

(c) Number distribution measured in 0.1M KNO_3 for a 4.34 mM (left, t=0d and t=5d) and 8.67 mM (right, t=5d) solution of **6**.

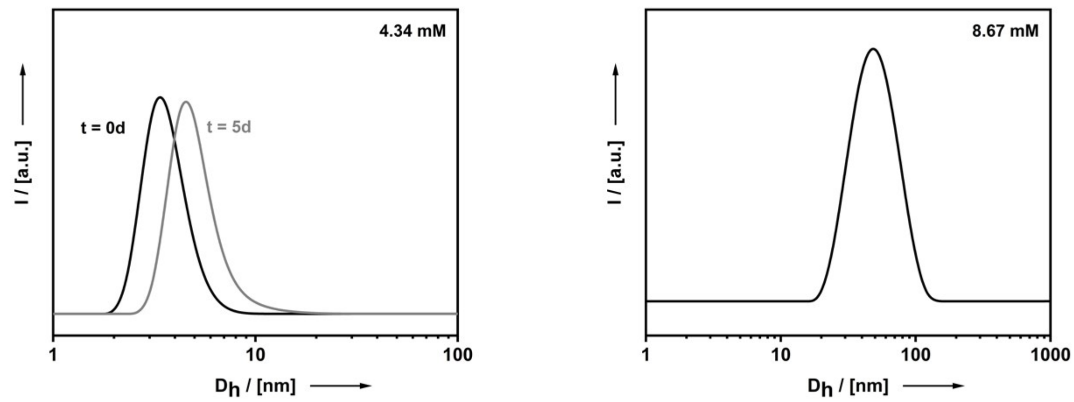

(d) Number distribution measured in 0.01M KPF_6 for a 14.81 mM solution of **6**.

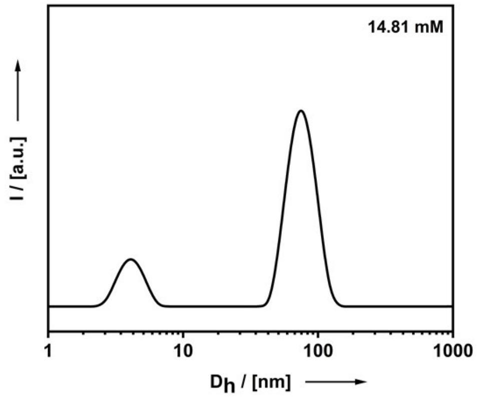

(e) DLS data of **6** measured in 0.01M KPF_6 for a temperature of 20°C (black) and 50°C (grey). Equilibration time per sample was 10 min.

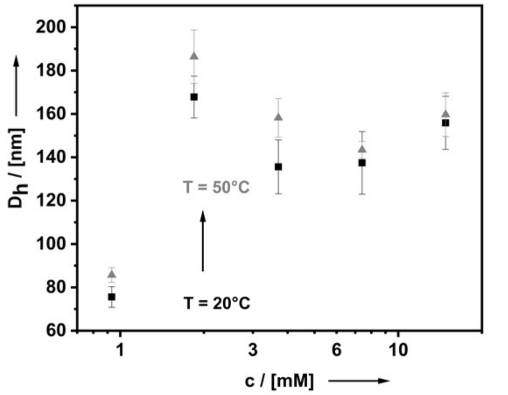

(f) Lyotropic liquid crystals of **6** in 0.01M KPF_6 solution.

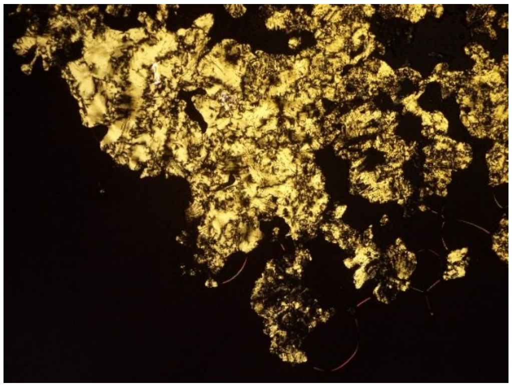

Fig. S9: EELS of **6**.

(a) EELS of **6**, energy loss specific for Fe_{L3}.

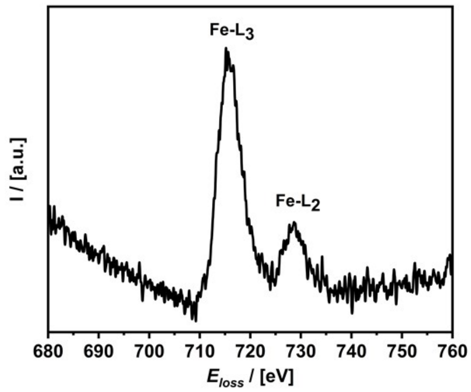

(b) EELS of **6**, energy loss specific for $O_{K\text{-edge}}$.

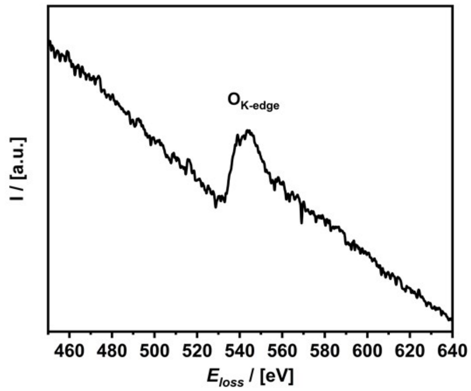

Fig. S10: Oxidation of **6** to **6⁺** and characterization of **6⁺**.

(a) Coloration of **6⁺**.

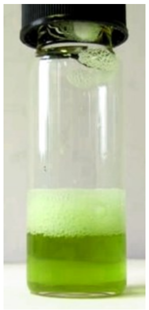

(b) UV-Vis spectrum of **6⁺** recorded in water.

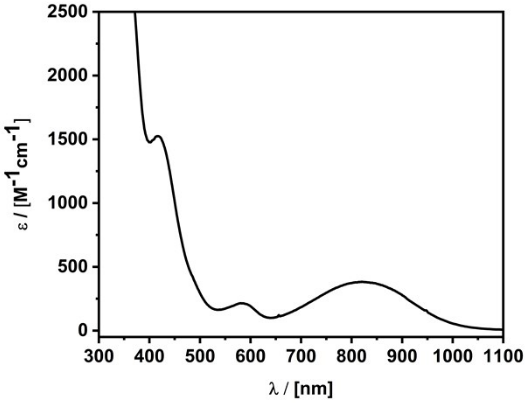

(c) Chemical oxidation ($(NH_4)_2[Ce(NO_3)_6]$) and rereduction (NaS_2O_3) of **6/6⁺**. ^{1}H NMR of **6** after rereduction measured in MeOD (Top). Compound **6** was extracted using NBu_4PF_6 (^{1}H NMR of NBu_4PF_6, bottom).

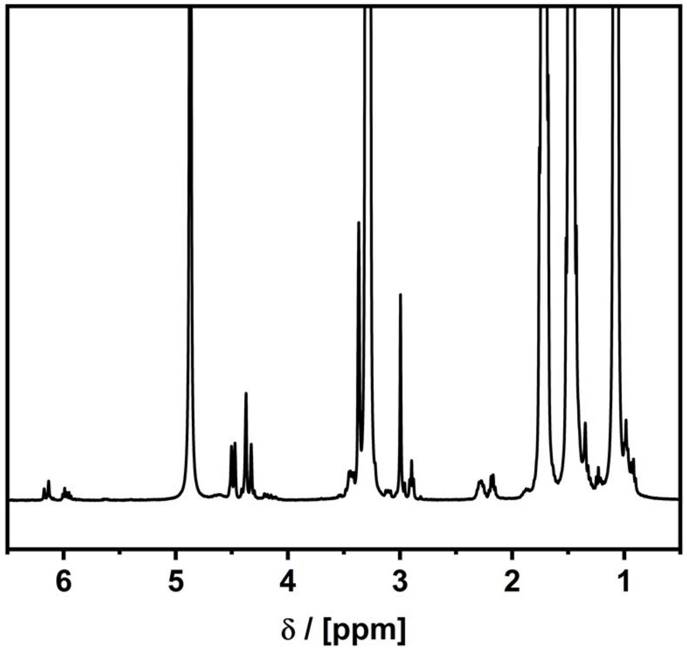

^{1}H NMR (400 MHz, MeOD) *δ* [ppm]: 6.15 (d, $^3J_{HH,trans}$ = 15.7 Hz, 1H, **H-12**), 5.97 (dt, $^3J_{HH,trans}$ = 15.0 Hz, $^3J_{HH}$ = 6.9 Hz, 1H, **H-13**), 4.50 (vt, $^3J_{HH}$ = 1.9 Hz, 2H, **H-7**), 4.47 (vt, $^3J_{HH}$ = 2.0 Hz, 2H, **H-10**), 4.40 – 4.35 (m, 4H, **H-8**, **H-5**), 4.33 (vt, $^3J_{HH}$ = 1.9 Hz, 2H, **H-9**), 3.45-3.39 (m, 2H, **H-3**), 3.00 (s, 6H, **H-4**), 2.90 (t, $^3J_{HH}$ = 6.9 Hz, 2H, **H-1**), 2.27 (p, $^3J_{HH}$ = 7.1 Hz, 2H, **H-2**), 2.23 – 2.17 (m, 2H, **H-14**), 0.96 (t, $^3J_{HH}$ = 7.1 Hz, 3H, **H-18**).

H-15, **H-16**, **H-17** are superimposed by NBu_4PF_6 proton signals.

^1H NMR spectrum of NBu_4PF_6.

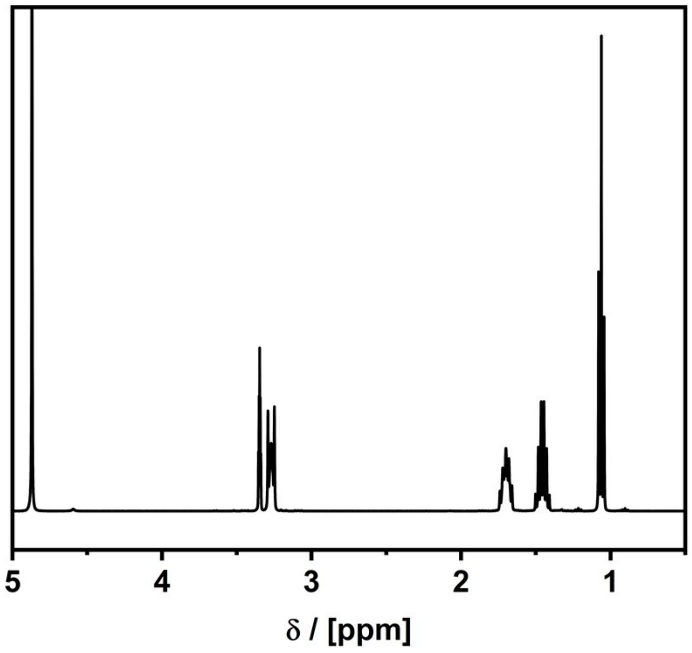

^{1}H NMR (400 MHz, MeOD) *δ* [ppm]: 3.31 – 3.23 (m, 8H), 1.70 (tt, $^3J_{HH}$ = 8.2, 6.1 Hz, 8H), 1.46 (h, $^3J_{HH}$ = 7.4 Hz, 8H), 1.06 (t, $^3J_{HH}$ = 7.3 Hz, 12H).

Fig. S11: Surfactant properties of **6⁺**.

(a) Surface tension measurement of **6⁺** performed in 0.1M aqueous KNO_3 solution.

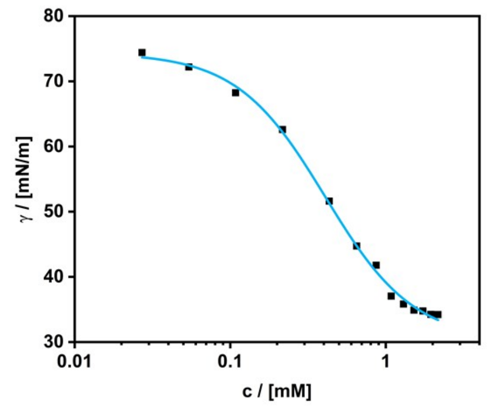

(b) DLS measurement of **6⁺** performed in 0.1M aqueous KNO_3 solution directly after preparation.

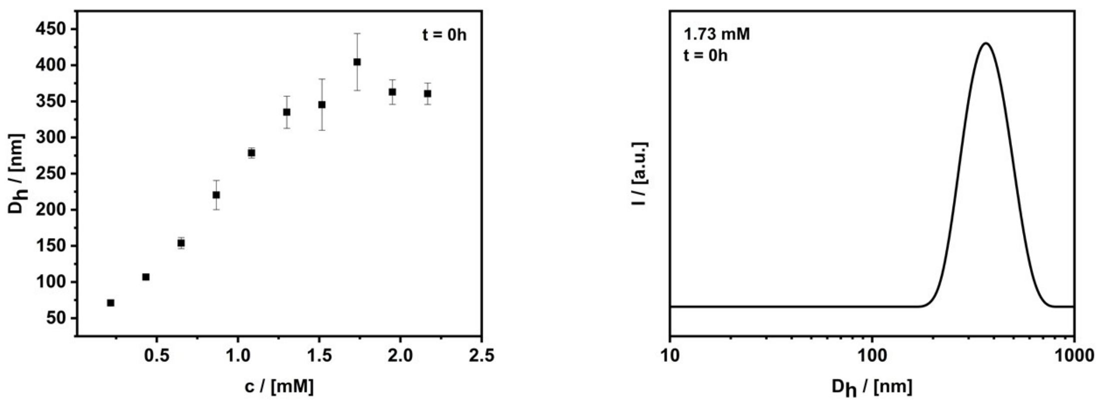

(c) DLS measurement of **6⁺** performed in 0.1M aqueous KNO_3 solution after t=2h.

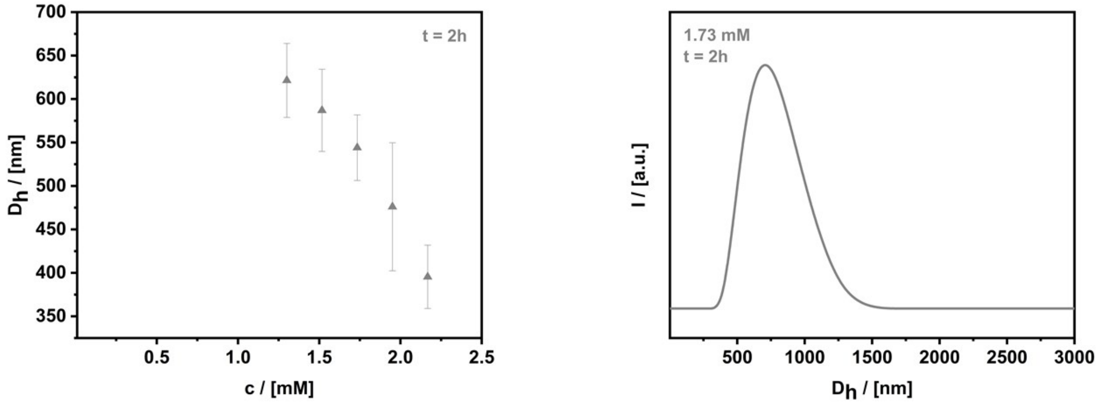

(d) Comparison of the DLS measurement of **6⁺** performed in 0.1M aqueous KNO_3 solution directly after preparation (t=0h) and after t=2h.

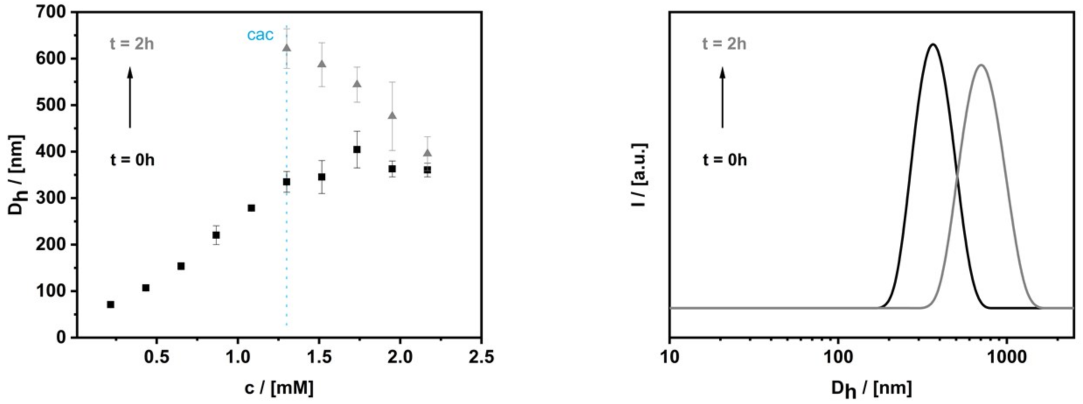

Fig. S12: EELS of **6⁺**.

(a) EELS of **6⁺**, energy loss specific for Fe_{L3}.

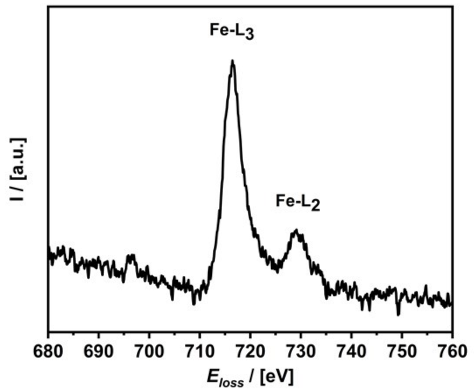

(b) EELS of **6⁺**, energy loss specific for $O_{K\text{-}edge}$.

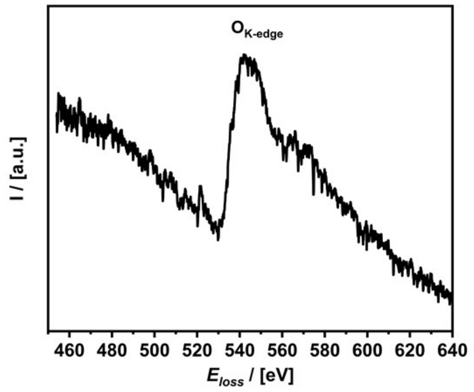

Fig. S13: Additional Cryo-TEM images of **6⁺** after exposure to the magnetic field

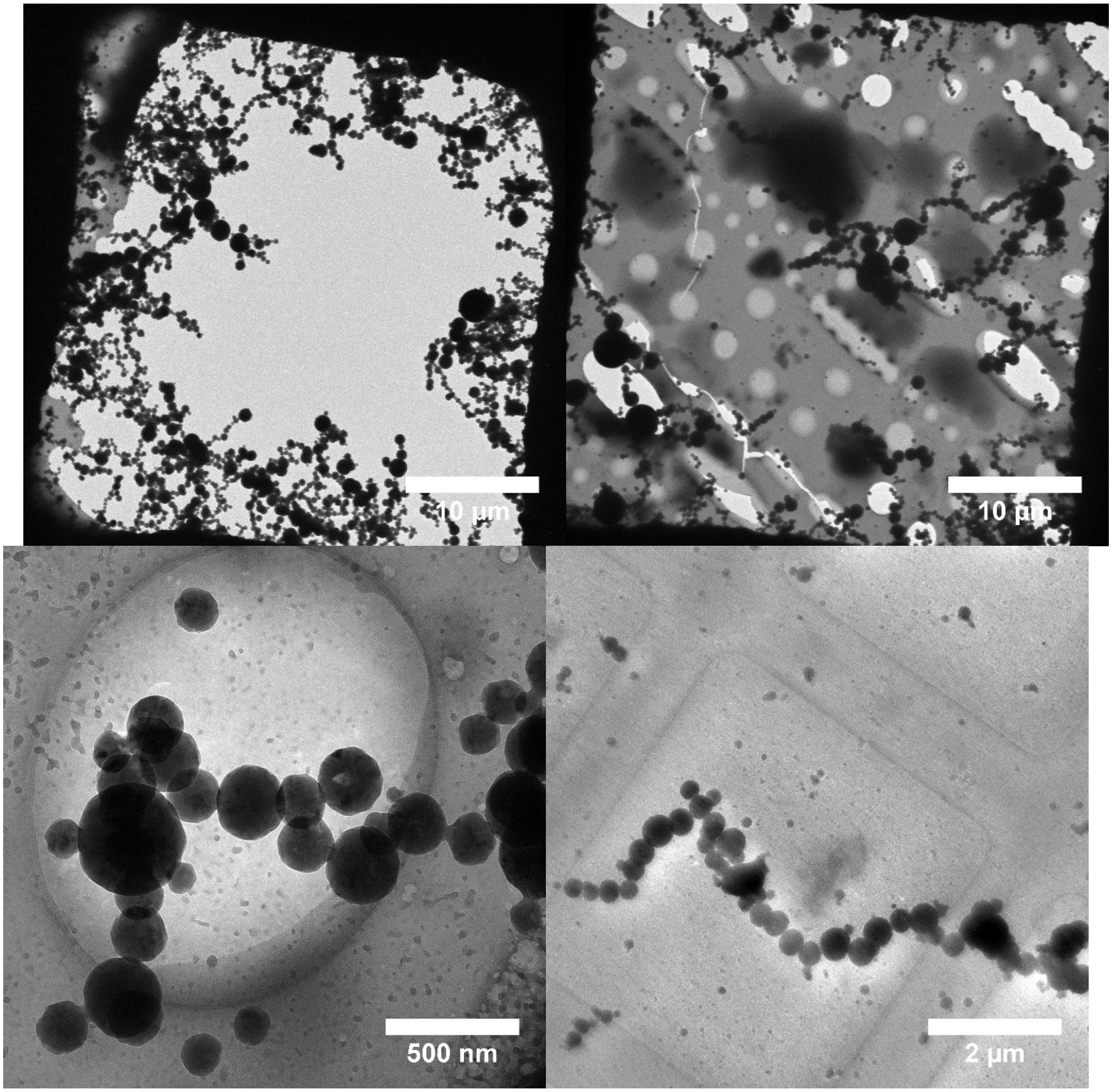

Fig. S14: Diffusion anisotropy factor of **6⁺** obtained from DLS orthogonal and parallel to the external magnetic field

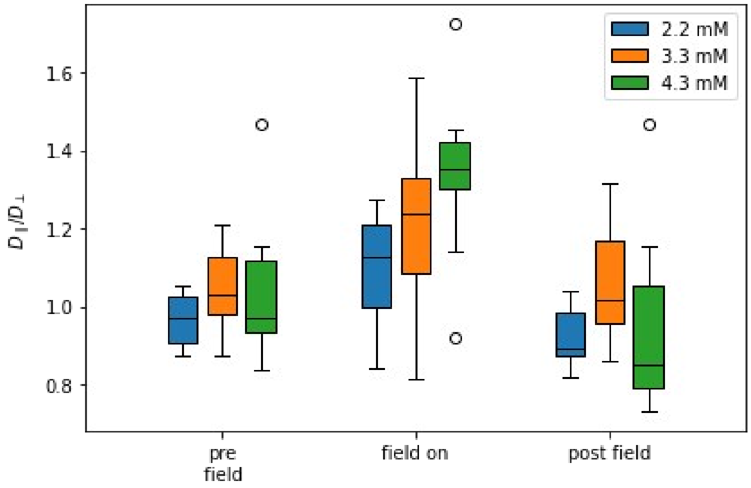

The diffusion coefficient was measured with DLS, parallel and perpedicular to the magnetic field. The ratio $D_\parallel$ / $D_\perp$ is shown for measurements before, while and after application of the external field (0.8T, 10 min). The effect of the external field is an increases of $D_\parallel$ / $D_\perp$, especially for the high concentration. This is in accordance with assuming chains of aggregates that align parallel to the field. The theorie predicts a factor of 2.0 for an ideal long rod.[4] Thus, the observed ratio of around 1.5 indicates a considerable anisotropy of the system. Each boxplot is generated from measurements at the absolute time 4:20, 4:40 and 5:00 after oxidation (see Fig. 6).

References

1. Stejskal, E. O.; Tanner, J. E., Spin Diffusion Measurements: Spin Echoes in the Presence of a Time-Dependent Field Gradient. *J. Chem. Phys.* **1965,** *42* (1), 288-292.
2. Tanner, J. E., Pulsed Field Gradients for NMR Spin-Echo Diffusion Measurements. *Rev. Sci. Instrum.* **1965,** *36* (8), 1086-1087.
3. Stejskal, E. O., Use of Spin Echoes in a Pulsed Magnetic-Field Gradient to Study Anisotropic, Restricted Diffusion and Flow. *J. Chem. Phys.* **1965,** *43* (10), 3597-3603.
4. Feldman, D., The theory of polymer dynamics, by M. Doi and S. F. Edwards, the Clarendon Press, Oxford University Press, New York, 1986, 391 pp. Price: $78.50. *J. Polymer. Sci., Part C: Polymer Lett.* **1989,** *27* (7), 239-240.
